# SPARK-X: non-parametric modeling enables scalable and robust detection of spatial expression patterns for large spatial transcriptomic studies

**DOI:** 10.1186/s13059-021-02404-0

**Published:** 2021-06-21

**Authors:** Jiaqiang Zhu, Shiquan Sun, Xiang Zhou

**Affiliations:** 1 Department of Biostatistics, University of Michigan, Ann Arbor, MI 48109 USA; 2 Center for Statistical Genetics, University of Michigan, Ann Arbor, MI 48109 USA; 3 Department of Epidemiology and Biostatistics, Xi’an Jiaotong University, Xi’an, Shaanxi 710061 P.R. China

**Keywords:** Spatial transcriptomics, SE analysis, Covariance test, Non-parametric modeling, Slide-seq, HDST, SPARK, SPARK-X, Spatial expression pattern

## Abstract

Spatial transcriptomic studies are becoming increasingly common and large, posing important statistical and computational challenges for many analytic tasks. Here, we present SPARK-X, a non-parametric method for rapid and effective detection of spatially expressed genes in large spatial transcriptomic studies. SPARK-X not only produces effective type I error control and high power but also brings orders of magnitude computational savings. We apply SPARK-X to analyze three large datasets, one of which is only analyzable by SPARK-X. In these data, SPARK-X identifies many spatially expressed genes including those that are spatially expressed within the same cell type, revealing new biological insights.

**Supplementary Information:**

The online version contains supplementary material available at 10.1186/s13059-021-02404-0.

## Background

Spatially resolved transcriptomic studies perform gene-expression profiling with spatial localization information on tissues and cell cultures [1–3]. These studies allow us to examine the spatial expression patterns of genes on the tissue [4–6], characterizing local structures and microenvironments [7, 8] and detecting cell-cell interactions across spatial locations [9, 10]. Spatial transcriptomic studies are enabled by various spatial transcriptomic technologies that are rapidly evolving. While early spatial transcriptomic technologies are often small in scale with relatively low spatial resolution [11, 12], recent technologies, such as Slide-seq [13, 14] and high-definition spatial transcriptomics (HDST) [15], have enabled transcriptome-wide profiling at micron resolution on tens or hundreds of thousands of spatial locations. The resulting large-scale spatial transcriptomic data, limited by sequencing depth, are also in sparse forms, with a prevalence of low counts and a substantial fraction of zero values (Additional file 1: Table S1). The sheer scale of these recent spatial transcriptomic data, paired with their sparse form, has created enormous computational and statistical challenges for many spatial transcriptomic analytic tasks.

A key analytic task in spatial transcriptomic studies is to identify genes that display spatial expression patterns, commonly referred to as SE genes. Such SE analysis is an important first step towards characterizing the spatial and functional organization of complex tissues [16, 17]. Common methods for performing SE analysis include trendsceek [4], SpatialDE [5], and SPARK [6]. Unfortunately, even the latter two computationally efficient methods are not readily applicable for analyzing large-scale sparse spatial transcriptomic data that are being collected today [6, 18]. Specifically, the computational complexity of both SpatialDE and SPARK scales cubically with respect to the number of spatial locations. Consequently, it would take days to months for either method to analyze large-scale spatial transcriptomic data. Similarly, the memory requirement of both SpatialDE and SPARK also scales cubically with respect to the number of spatial locations. Analyzing large-scale spatial transcriptomic data by either method would require dozens to thousands of GB physical RAM memory, which can be challenging to satisfy even on large computing clusters [19]. Finally, the large-scale spatial transcriptomic data are often in the form of sparse counts with a prevalence of zero values. While the large fraction of zeros is not due to dropout events and can be effectively accounted for by an over-dispersed Poisson distribution (Additional file 1: Figure S14), such sparse data is nevertheless challenging to model parametrically. Specifically, direct modeling of sparse count data with a negative binomial distribution or other over-dispersed Poisson distributions incurs algorithm stability issues [6, 20, 21], and, as will be shown below, can lead to a failure of convergence in more than 90% of genes in large-scale spatial transcriptomic data. On the other hand, approximate modeling of sparse count data by a Gaussian distribution as in SpatialDE and the Gaussian version of SPARK is not ideal either [22, 23], as such parametric approximation leads to both power loss and failure of type I error control at small *P* values that are essential for detecting SE genes at the transcriptome-wide significance level.

Here, we present SPARK-X (SPARK-eXpedited), a scalable non-parametric test for SE analysis, that addresses the aforementioned challenges. SPARK-X builds upon a robust covariance test framework [24–27] and extends it to incorporating a variety of spatial kernels for non-parametric spatial modeling of sparse count data from large spatial transcriptomic studies. With additional algebraic innovations, SPARK-X reduces computational complexity and physical RAM memory requirement for SE analysis from cubic to linear with respect to the number of spatial locations, resulting in several orders of computational speed improvements and several orders of physical RAM memory savings as compared to existing approaches. Importantly, due to its non-parametric nature, SPARK-X is algorithmically stable and statistically robust with respect to the underlying data generative process, providing calibrated type I error control and improved power across a range of data types collected through a variety of technical platforms. We illustrate the benefits of SPARK-X via applications to three large-scale spatial transcriptomic data collected by different technologies, one of which is only analyzable by SPARK-X. In the analysis, we identified many new SE genes including those that display spatial expression pattern within the same cell type. These SE genes are involved in synaptic organization and functional compartmentalization of the cerebellum and involved in lateral inhibition and odor discrimination in the olfactory system.

## Results

### Simulations

**Fig. 1:**
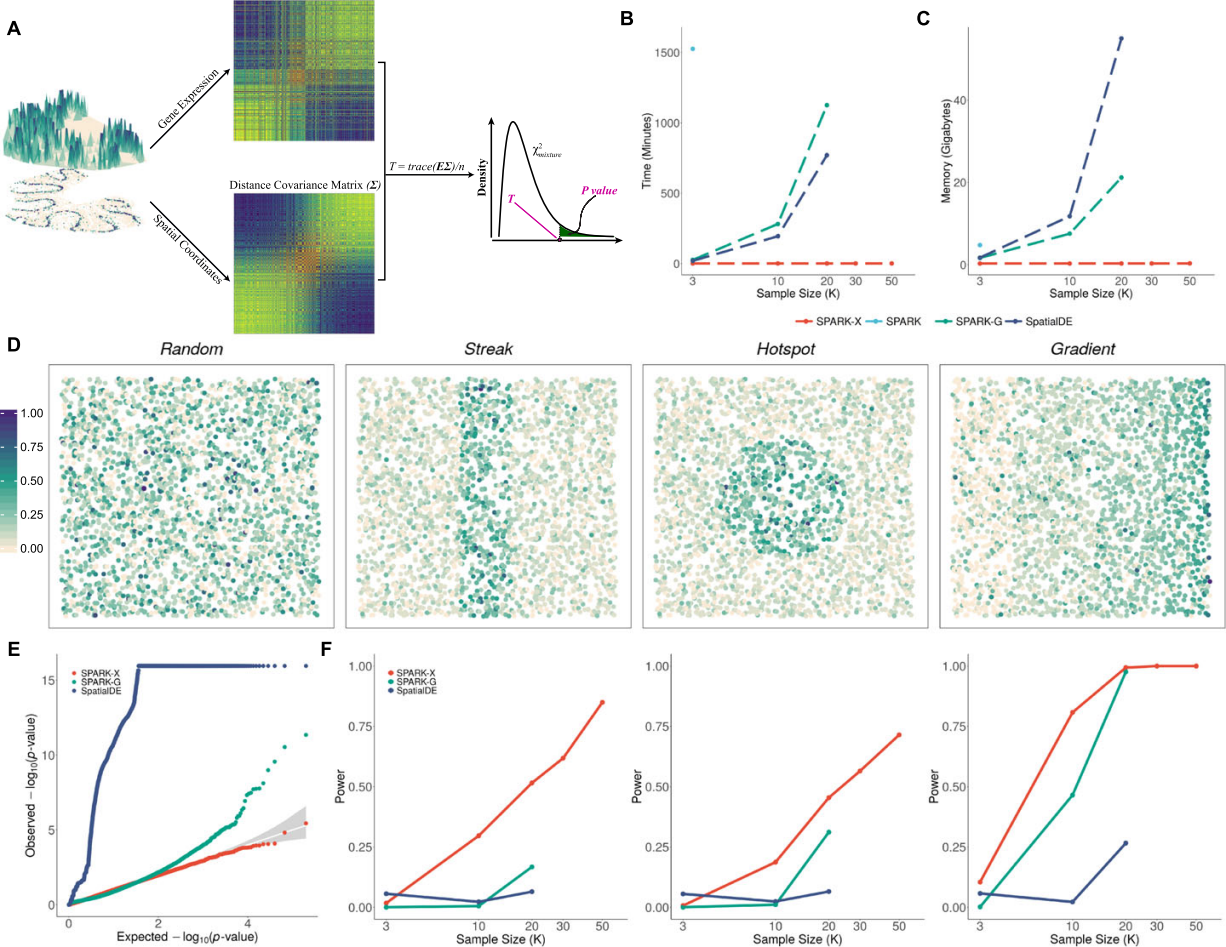
Method schematic of SPARK-X and simulation results. **A** Schematic of the SPARK-X method. **B** Computational time of different methods for analyzing data with different sample sizes in the simulations. Plot shows computational time in minutes (y-axis) for analyzing 10,000 genes with different sample sizes (x-axis) for different methods. Compared methods include SPARK-X (red), SPARK (sky blue), SPARK-G (green) and SpatialDE (steel blue). **C** Random access memory (RAM) of different methods for analyzing data with different sample sizes in the simulations. Plot shows computational memory in gigabytes (y-axis) for analyzing 10,000 genes with different sample sizes (x-axis) for different methods. Computations are carried out using a single thread of an Intel Xeon E5-2683 2.00 GHz processor. SPARK-X is much more computationally efficient than SPARK, SPARK-G, and SpatialDE. For ease of computation, we did not apply SPARK to the data with sample size greater than 3000 and did not apply SPARK-G and SpatialDE to the data with sample size greater than 30,000. **D** Representative genes displaying random pattern and other three spatial expression patterns. **E** Quantile–quantile plot of the observed −log_10_(*P*) from different methods against the expected −log_10_(*P*) under the null simulations with high sparsity (*μ* = 0.005). *P* values were combined across ten simulation replicates. Simulations were performed under moderate sample size (n = 10,000) and moderate dispersion (2.5). **F** Power plots show the proportion of true positives (y-axis) detected by different methods at a range of sample sizes (x-axis) for the alternative simulations with high sparsity at an FDR cutoff of 0.05. Simulations were performed under a moderate fraction of marked cells (20%) and moderate SE strength (threefold) for the hotspot and streak patterns or under moderate SE strength (30% cells displaying expression gradient) for the gradient pattern

A method schematic of SPARK-X is shown in Fig. 1A, with details provided in the “Methods” section. We performed realistic simulations (Additional file 1: Figure S16A) to evaluate the performance of SPARK-X and compare it with three existing approaches, the Poisson version of SPARK (SPARK), the Gaussian version of SPARK (SPARK-G) and SpatialDE. Simulation details are provided in the “Methods”. Briefly, in each scenario, we generated coordinates for a fixed number of spatial locations through a random-point-pattern Poisson process and simulated 10,000 genes on the spatial locations based on a negative binomial distribution using parameters inferred from real data. We examined both type I error control under the null hypothesis and power for identifying SE genes under various alternatives. In the null simulations, all genes are non-SE genes with expression levels randomly distributed across spatial locations without any spatial patterns (Fig. 1D). In the alternative simulations, 9000 genes are non-SE genes, while 1000 genes are SE genes whose expression levels display one of the three spatial patterns (hotspot, streak and gradient; Fig. 1D). Because some methods fail to control for type I error, we measured power in the alternative simulations based on false discovery rate (FDR) to ensure fair comparison among methods—though *P* values from SPARK-X are well calibrated across scenarios and thus can be directly used in place of FDR for declaring significance. In the simulations, we varied the number of samples; varied the sparsity of the data to be moderate (average 62.1% zeros; similar to early spatial transcriptomics data) or high (average 99.5% zeros; similar to recent Slide-seq data); varied SE strength for SE genes to be either weak, moderate, or strong; and varied a set of other relevant parameters.

In the null simulations, we found that SPARK-X produces well-calibrated *P* values at transcriptome-wide significance levels (Fig. 1E and Additional file 1: Figure S2B), regardless of the data sparsity level. When data sparsity is moderate, both SPARK and SPARK-G yield reasonably calibrated *P* values as observed in previous studies [6]. However, when data sparsity is high, SPARK fails to converge for all genes while SPARK-G produces inflated *P* values. The failure of SPARK for sparse data depends on the sparsity level of the input data and is presumably due to the numerical instability of the penalized quasi-likelihood algorithm when the outcome consists of low read counts with a high percentage of zero values. Such algorithmic issue is not restricted to SPARK and appears to be general for fitting algorithms of the generalized linear models such as the negative binomial model (Additional file 1: Table S2). The failure of SPARK-G in controlling for type I error in sparse data is presumably due to the inaccurate approximation of sparse count data with a Gaussian distribution and the fact that the variance stability transformation can no longer properly remove the correlation between mean and variance there [28]. SpatialDE produces overly conservative *P* values when data sparsity is moderate and unduly inflated *P* values when sparsity is high. The failure of SpatialDE in controlling type I errors in these settings is presumably due to its Gaussian modeling of count data, use of an asymptotic test in place of an exact test, and/or use of an *ad hoc* minimal *P* value combination rule. The *P* value calibration results in terms of genomic inflation factor for different methods are consistent across a range of sample sizes (Additional file 1: Figure S1).

In the alternative simulations, we found that SPARK-X is as powerful as SPARK when data sparsity is moderate (Additional file 1: Figure S2C) and is more powerful than all other methods when data sparsity is high (Fig. 1F). Specifically, when data sparsity is moderate, both SPARK-X and SPARK are more powerful than SPARK-G and SpatialDE in detecting streak and hotspot patterns, regardless of sample size. SPARK-X, SPARK-G, and SPARK are more powerful than SpatialDE in detecting the gradient pattern when the sample size is small, though all four methods have comparable power when the sample size is moderate or large. When data sparsity is high, SPARK-X is more powerful than both SPARK-G and SpatialDE, while SPARK fails to converge for all genes. The power gain by SPARK-X over SPARK-G and SpatialDE for sparse data increases with increasing sample size. The power comparison results hold across a range of simulation settings (Additional file 1: Figures S3 and S4), highlighting the robust performance of SPARK-X for analyzing large sparse spatial transcriptomic data.

Importantly, SPARK-X is much more computationally efficient than the other methods, with orders of magnitude improvement in terms of computation time and memory requirement (Fig. 1B, C). For example, it takes SPARK-G and SpatialDE 1125 and 770 min, respectively, to analyze a data with 10,000 genes and 20,000 spatial locations. In contrast, it only takes SPARK-X 1 min to analyze the same data. Similarly, while SPARK-G and SpatialDE require 21.2 and 55.3 gigabytes (GB) of physical RAM memory, respectively, SPARK-X only requires 0.32 GB. The computation gain by SPARK-X is even more appreciable in data with larger samples. Indeed, with moderate computation resource, SPARK-X is the only method applicable to data with sample size exceeding around 30,000.

### SPARK-X enables powerful SE analysis in the Slide-seq cerebellum data

We applied SPARK-X along with SPARK-G and SpatialDE to analyze three published data obtained with three different spatial transcriptomic technologies: one by Slide-seq, one by Slide-seqV2, and the other by HDST (details in “Methods”). We did not apply the Poisson version of SPARK to analyze any of these data due to its excessive computational requirements there.

**Fig. 2:**
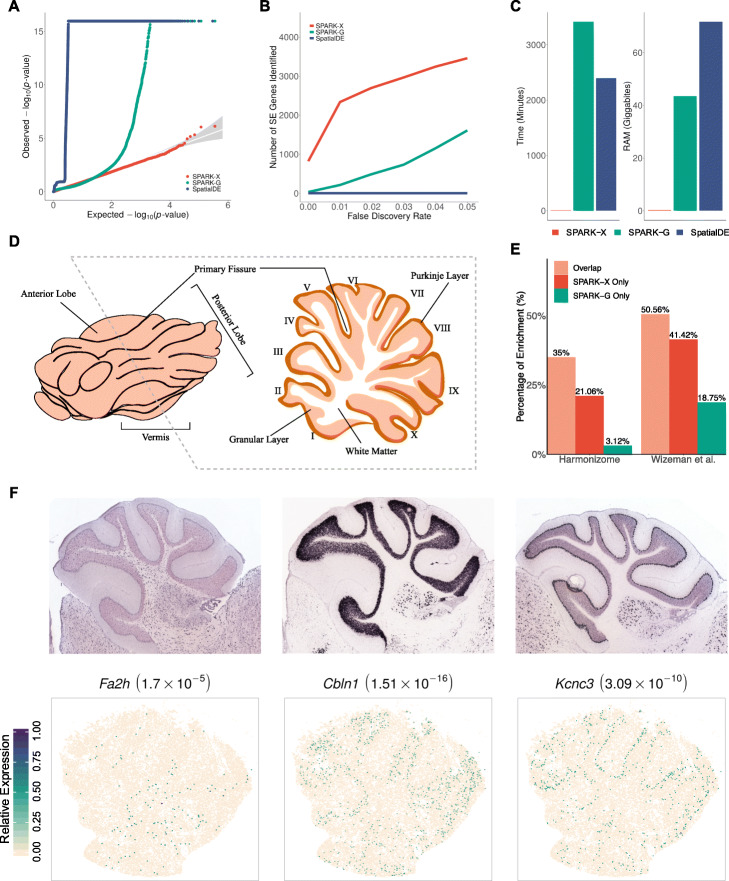
Analyzing the mouse cerebellum Slide-seq data. **A** Quantile–quantile plot of the observed −log_10_(*P*) from different methods against the expected −log_10_(*P*) under the null condition in the permuted Slide-seq data. *P* values were combined across ten permutation replicates. Compared methods include SPARK-X (red), SPARK-G (green) and SpatialDE (steel blue). **B** Power plot shows the number of genes with spatial expression pattern (y-axis) identified by different methods at a range of FDRs (x-axis) in the Slide-seq data. **C** Bar plots show the computation time and RAM usage of different methods for analyzing the mouse cerebellum Slide-seq data. **D** An illustration of the mouse cerebellum, where a cross-section shows its lobular organization. **E** Bar plot displays the percentage of SE genes identified by either SPARK-X (red) or SPARK-G (green) or both (orange) that were also validated in two gene lists: one from the Harmonizome database (left) and the other from literature (right; Wizeman et al). **F** Visualization of three representative SE genes identified only by SPARK-X in the Slide-seq data. The top panel shows in situ hybridization results for the three genes obtained from the Allen Brain Atlas. The bottom panel shows relative gene-expression levels (green, high; antique-white, low), with *P* values from SPARK-X displayed inside parentheses

The first data we examined is a mouse cerebellum data generated through Slide-seq [13], consisting of gene expression measurements for 17,729 genes on 25,551 beads. Consistent with simulations, we found that SPARK-X produced calibrated *P* values under permuted null, while SPARK-G and SpatialDE did not (Fig. 2A). Also consistent with simulations, SPARK identified more SE genes as compared to SPARK-G and SpatialDE across a range of empirical FDRs (Fig. 2B, Additional file 1: Figures S5 and S6). For example, at an FDR of 1%, SPARK-X identified 2336 SE genes, which is approximately ten times more than that detected by SPARK-G (which identified 212, among which 180 overlapped with SPARK-X; Additional file 1: Figure S5A). SpatialDE was unable to detect any SE genes in the data, consistent with its low power in large-scale sparse data as observed in the simulations.

We provided three lines of evidence to support the validity of SE genes detected by SPARK-X. First, we found that SE genes only detected by SPARK-X expressed on comparable number of spots as compared to the SE genes detected by both methods (Additional file 1: Figure S5A). In contrast, most SE genes only detected by SPARK-G appeared to be expressed on very few spots (Additional file 1: Figure S5A and S5C), suggesting potentially false signals. Second, we obtained a list of 2632 genes related to mouse cerebellum from the Harmonizome database [29]. Reassuringly, 22% of the unique SE genes identified by SPARK-X were in the Harmonizome list, while only 3% of the unique SE genes identified by SPARK-G were in the same list (Fig. 2E). Third, we obtained a list of 4152 cell type-specific genes identified in a recent single-cell RNA sequencing study in the mouse cerebellum [30]. Again, 41% of the unique SE genes identified by SPARK-X were in the marker list, while only 19% of the unique SE genes identified by SPARK-G were in the same list (Fig. 2E). These three validation analyses provide convergence support for the higher power of SPARK-X.

We performed functional enrichment analyses on SE genes detected by SPARK-X and SPARK-G (“Methods”). A total of 808 enriched Gene Ontology (GO) terms (Additional file 1: Figure S5B and Additional file 2: Table S4) and 328 Reactome pathways were identified based on SPARK-X SE genes, while only 223 GO terms (overlap = 115) and 56 Reactome pathways (overlap = 46) were identified based on SPARK-G SE genes. Many enriched GO terms identified only by SPARK-X were directly related to synaptic organization of the cerebellum. For example, one enriched GO term was peripheral nervous system development (GO 0007422; *P* = 1.57 × 10^− 3^). Its representative gene *Fa2h* encodes the fatty acid 2-hydroxylase and is highly enriched in oligodendrocytes (Fig. 2D, F). Fatty acid 2-hydroxylase is known to play an important role in synthesizing myelin galactolipids on oligodendrocytes and facilitates the subsequent myelination that is essential for axon protection and signal transduction [31]. Another enriched GO term was synapse organization (GO 0050808; *P* = 3.19 × 10^− 11^). Its representative gene *Cbln1* encodes a cerebellum-specific precursor protein precerebellin and is highly enriched in the granular layer as supported by previous in situ hybridization evidence [32] (Fig. 2F). Precerebellin is a unique synapse organizer for matching and maintaining pre- and post-synaptic elements between parallel fibers and Purkinje cells in the cerebellum and a key for the functional induction of long-term depression there [33]. As a last example, the GO term of neurotransmitter secretion regulation is only identified by SPARK-X (GO 0046928; *P* = 2.56 × 10^− 5^). Its representative gene *Kcnc3* encodes the Potassium voltage-gated channel subunit Kv3.3 and is enriched in the Purkinje cells (Fig. 2F). *Kcnc3* plays an important role in regulating the frequency, shape, and duration of action potentials in the Purkinje cells and facilitates motor coordination [34, 35]. Overall, the new SE genes and GO terms identified by SPARK-X reveal important spatial and functional organization of the cerebellum that are missed by other SE methods, highlighting the benefits of running SE analysis with SPARK-X.

### SPARK-X enables detection of SE genes not explained by cell types in the Slide-seqV2 cerebellum data

The second data we examined is another mouse cerebellum data generated through Slide-seqV2 [14], consisting of gene expression measurements for 20,117 genes on 11,626 beads. In the analysis, SPARK-X produced calibrated *P* values under permuted null, while SPARK-G and SpatialDE did not (Additional file 1: Figure S7A). SPARK-X identified 688 SE genes, which is approximately six times more than that detected by SPARK-G (which identified 112, among which 68 overlapped with SPARK-X; Additional file 1: Figure S7B). SpatialDE was unable to detect any SE genes in the data. Functional enrichment analyses on SE genes detected by SPARK-X identified 595 enriched GO terms and 61 Reactome pathways, many of which are again directly related to synaptic organization of the cerebellum (Additional file 1: Figure S7D and Additional file 3: Table S5).

**Fig. 3:**
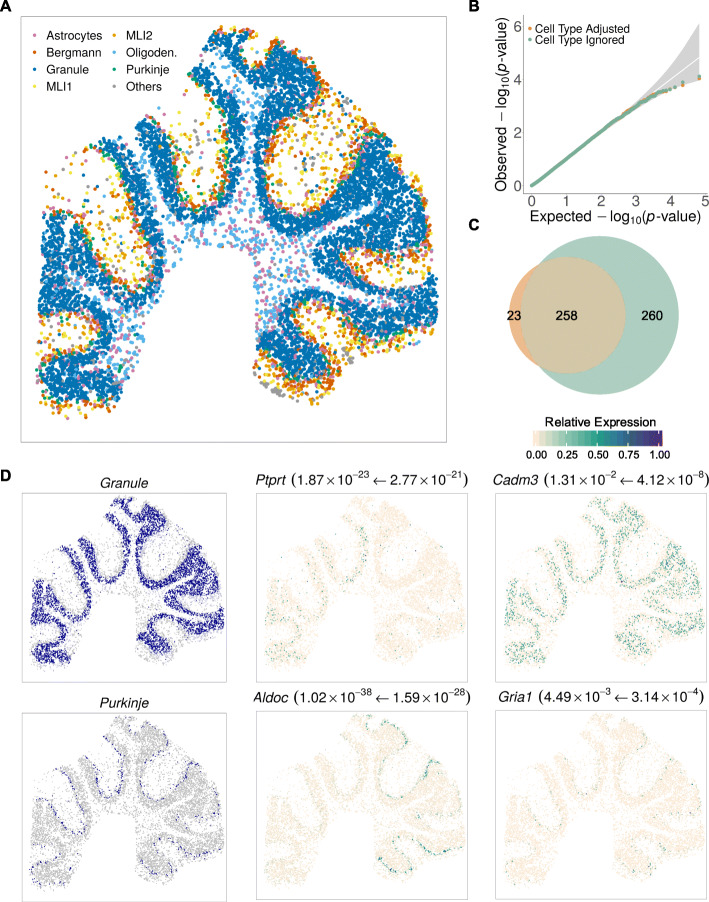
Analyzing the mouse cerebellum Slide-seqV2 data. **A** Spatial distribution of all major cell types in the Slide-seqV2 data. Cells are colored by cell types shown in the legend, where the cell type information were predicted using RCTD. **B** Quantile–quantile plot of the observed −log_10_(*P*) from different methods against the expected −log_10_(*P*) under the null condition in the permuted Slide-seqV2 data. *P* values were combined across ten permutation replicates. Permutations were run with SPARK-X with (tan) or without (pale green) adjusting for cell types. **C** Venn diagram shows the overlap in SE genes identified by SPARK-X with (tan) or without (pale green) adjusting for cell types. **D** Spatial distribution of predicted cell types (left two panels; blue represents cell type) and spatial expression pattern of four representative SE genes (middle and right panels; green represents high expression while antique-white represents low expression) in Slide-seqV2 data. *P* values from SPARK-X with (left side of the arrow) or without (right side of the arrow) adjusting for cell types are shown inside parentheses

One important feature of SPARK-X is its ability to control for covariates in the SE analysis. Such feature, when paired with the high sensitivity of Slide-seqV2 technology, provides us with a unique opportunity to investigate the extent to which SE genes display spatial expression pattern beyond those explained by spatial distribution of cell types. To do so, we inferred cell type compositions on majority of the spatial locations (82.2%) using RCTD [36] and treated the inferred compositions as covariates for SE analysis on these spatial locations (details in “Methods”; Fig. 3A and Additional file 1: Figure S8A). SPARK-X identified 281 and 518 SE genes with and without controlling for cell type compositions, respectively (overlap = 258, Fig. 3C), with calibrated *P* values under permuted null in the corresponding analyses (Fig. 3B). The result suggests that approximately half of the SE genes can be accounted for by the spatial distribution of cell types, consistent with our parallel analysis result that 46.7% SE genes were cell type markers identified in a recent single-cell RNA sequencing study in the mouse cerebellum [30]. For example, cell type marker genes, such as *Cadm3* and *Gria1* (Fig. 3D), were no longer SE genes conditional on the cell type composition, suggesting that their spatial expression patterns were primarily driven by the spatial distribution of the corresponding cell types. On the other hand, SE genes such as *Ptprt* and *Aldoc* remained significant after controlling for cell type compositions (Fig. 3D and Additional file 1: Figure S8B). A careful examination shows that *Ptprt* is highly expressed in a subset of granule cells in the anterior lobe of the cerebellum while *Aldoc* is highly expressed in a subset of Purkinje cells in the posterior lobe (Fig. 3D). Such distinctive and complementary spatial expression patterns of *Ptprt* and *Aldoc* in the anterior versus posterior lobe highlight the regional specification and functional compartmentalization of the cerebellum. *Ptprt* encodes the protein tyrosine phosphatase receptor rho (PTP*ρ*) that regulates synapse formation through interacting with cell adhesion molecules [37]. The expression pattern of *Ptprt* coincides with the granule cell lineage boundary between the anterior and posterior lobules around lobule VI [38, 39], supporting its potential role in the function of granule cells in sensorimotor transmission that is specialized in the anterior cerebellar cortex [40]. On the other hand, *Aldoc* encodes aldolase C, which is a brain-specific glycolytic isozyme and a well-known cerebellum compartmentation maker. *Aldoc* is expressed in Purkinje cells in a longitudinal striped fashion in the cerebellum. Each *Aldoc* expressed stripe receives enhanced glutamatergic innervations from climbing fibers originated from specific subnuclei of the inferior olive and projects to distinct subdivision of the deep cerebellar nuclei that further sends inhibitory projections back to the inferior olive [41–43]. Each *Aldoc* strip thus represents an anatomically connected olivocerebellar-nuclear module, with highly synchronous neuronal activity observable within each module and asynchronous activity between modules [44]. Notably, both *Ptprt* and *Aldoc* were also detected as SE genes, along with many others, in a cell type SE specific analysis where we applied SPARK-X to a subset of spatial locations that are dominated by either Purkinje cells or granule cells to directly detect genes that display spatial expression pattern within a cell type (Additional file 1: Figure S9). Overall, the structural and functional compartmentalization in the cerebellum revealed by cell type adjusted SE analysis highlights the utility of SPARK-X.

### SPARK-X enables scalable SE analysis of the HDST olfactory bulb data

**Fig. 4:**
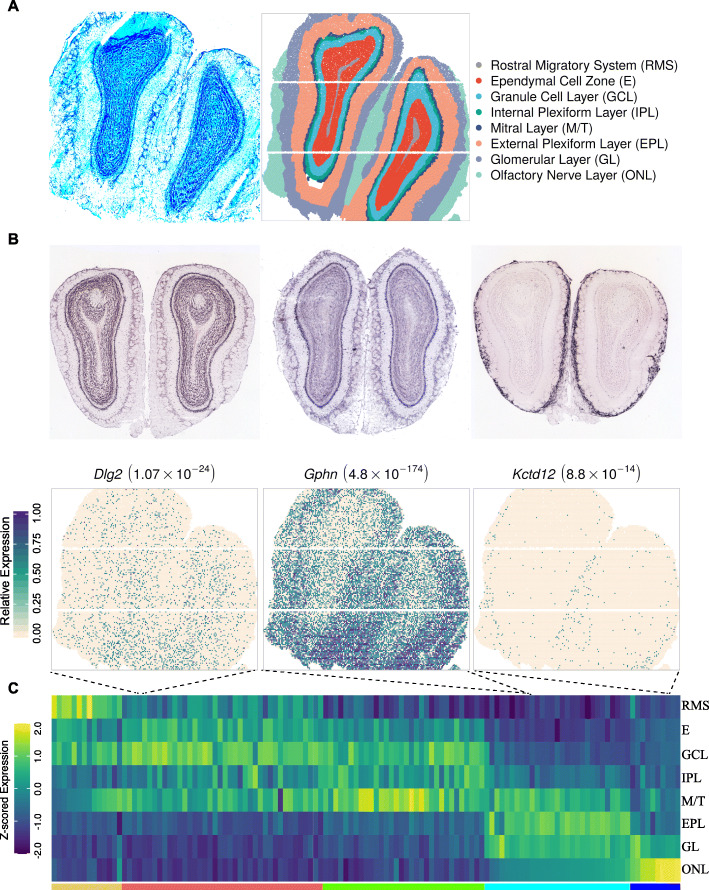
Analyzing mouse olfactory bulb HDST data. **A** H&E image of mouse olfactory bulb (left panel) and matching morphological annotation (middle panel) in the HDST data. Spots are colored by morphological layer shown in the legend (right panel), where the layer annotations were from ref [15]. H&E image reproduced from ref. [15] with permission. **B** Visualization of three representative SE genes identified only by SPARK-X in the HDST data. The top panels show in situ hybridization of the three genes obtained from the Allen Brain Atlas. The bottom panels show spatial expression patterns of the three genes at single-cell-level resolution (green, high expression; antique-white, low expression). *P* values from SPARK-X are shown inside parentheses. **C** Heatmap shows expression level of 125 SE genes (columns) across eight major morphological layers (rows). Colored bar at the bottom represents five gene clusters. RMS, rostral migratory system; E, ependymal cell zone; GCL, granule cell layer; IPL, internal plexiform layer; M/T, mitral layer; EPL, external plexiform layer; GL, glomerular layer; ONL, olfactory nerve layer

SPARK-X provides substantial computational gains over the other methods. For example, in the first data, it took SPARK-X 3 min to analyze the whole data, while it took 56 h for SPARK-G and 47 h for SpatialDE, respectively (Fig. 2C and Additional file 1: Table S1). In the second data, it took SPARK-X 2 min to analyze the whole data, while it took 13 h for SPARK-G and 8 h for SpatialDE, respectively (Additional file 1: Figure S7C and Table S1). The computational gain of SPARK-X becomes even more apparent in the third data, which is a mouse olfactory bulb data collected through HDST, consisting of 19,913 genes measured on 177,455 spots (Fig. 4A). This data is particularly challenging for existing SE methods due to the large number of spots measured there. Specifically, it would take an estimated 114 and 80 days if we use SPARK-G and SpatialDE to analyze the data. These two methods would also require 2100 and 3500 GB of memory, respectively (Additional file 1: Table S1). The high computational requirements for SPARK-G and SpatialDE thus exclude their use in the data. In contrast, SPARK-X requires 0.42 GB memory and 3 min of computing time and is the only SE method applicable to the data.

In the analysis, SPARK-X identified a total 125 SE genes, with calibrated *P* values under permuted null (Additional file 1: Figure S10A). Almost all SE genes showed clear spatial expression patterns that were cross validated by in situ hybridization in the Allen Brain Atlas [45] (Fig. 4B and Additional file 1: Figure S10E). The 125 SE genes are clustered into five clusters that were enriched in distinct morphological layers of the olfactory bulb (Fig. 4C and Additional file 1: Figure S10C). Functional enrichment analyses identified 377 enriched GO terms and 39 Reactome pathways, many of which are related to synaptic organization and signaling (Additional file 1: Figure S10D and Additional file 4: Table S6). For example, one enriched GO term is synaptic membrane (GO:0097060, *P* = 2.50 × 10^− 15^), with a representative gene *Kctd12*. *Kctd12* encodes an auxiliary GABA_B_ receptor subunit [46] and is observed to be highly expressed in the olfactory nerve layer (Fig. 4B, C), consistent with its enrichment in the glomerulus [47]. Another enriched GO term is the GABA-ergic synapse (GO: 0098982, *P* = 1.19 × 10^− 5^) with a representative gene *Gphn*. *Gphn* encodes the protein gephyrin and is observed here to be highly expressed in the mitral layer and external plexiform layer. Similar to GABA-ergic synapse, glutamatergic synapse (GO: 0098978, *P* = 3.35 × 10^− 15^) is also enriched. The representative gene of glutamatergic synapse, *Dlg2*, encodes a membrane-associated guanylate kinase and is observed here to be highly expressed in both the granule cell layer and the mitral layer. The complementary expression pattern of inhibitory GABA-ergic and excitatory glutamatergic neurons as represented by *Gphn* and *Dlg2* are consistent with the spatial organization of lateral inhibition: mitral cells activate granule cells that in turn inhibits nearby mitral cells, leading to robust odor processing and discrimination in the olfactory bulb [48–52].

We performed conditional analysis to investigate the extent to which SE genes display spatial expression pattern beyond those explained by the spatial distribution of cell types. Specifically, we extracted 103,602 spots with confident cell type assignment in the original study (Additional file 1: Figure S11D) and treated the assigned cell types as covariates for SE analysis on these spatial locations (details in “Methods”). SPARK-X identified 36 and 66 SE genes with and without controlling for cell types, respectively (overlap = 35, Additional file 1: Figure S11C), with calibrated *P* values under the permuted null in the corresponding analyses (Additional file 1: Figure S11A). The results suggest that more than half of the SE genes can be accounted for by the spatial distribution of cell types, consistent with our parallel analysis result that 59.1% SE genes were cell type markers identified in a recent single-cell RNA sequencing study in the mouse olfactory bulb [53]. Careful examination of the detected SE genes suggests that genes that remained significant after controlling for cell types often display spatial expression pattern across multiple cell types or within the same cell type (Additional file 1: Figure S11E and S11F). For example, *Camk1d* is enriched in the ventral part of multiple olfactory bulb layers including the external plexiform layer and the glomerular layer. *Camk1d* is also detected as an SE gene when we applied SPARK-X to a subtype of inhibitory neurons, the olfactory bulb inner horizontal cells, to directly detect SE genes that display spatial expression pattern within the cell type. In particular, *Camk1d* is specifically enriched in a subset of these inhibitory neurons that reside outside the granule cell layer (Additional file 1: Figure S11F). As another example, *Kcnip1* is also an SE gene that is detected in both conditional analysis and cell type-specific analysis. *Kcnip1* displays similar spatial expression pattern as the *Camk1d* and its *P* value becomes slightly more significant after controlling for cell types (Additional file 1: Figure S11E). *Camk1d* encodes the calcium/calmodulin-dependent protein kinase that operates in the calcium-triggered CaMKK-CaMK1 signaling cascade [54] and *Kcnip1* encodes the cytosolic voltage-gated potassium channel-interacting protein that regulates neuronal membrane excitability [55]. Their known roles in regulating signaling pathways in inhibitory neurons, paired with their restricted spatial expression in a subset of inhibitory neurons in the external plexiform and glomerular layers, suggest their potential involvement in lateral inhibition in the olfactory bulb.

## Discussion

We have presented a new method, SPARK-X, for identifying SE genes in large-scale sparse spatial transcriptomic data. In comparison to existing approaches, SPARK-X is highly computationally efficient, produces well-calibrated *P* values and ensures robust performance for large spatial transcriptomic data sets collected across a range of technologies. The modeling framework of SPARK-X is also flexible, allowing for potential future extensions towards transcriptome-wide joint modeling of correlated genes. We have illustrated the benefits of SPARK-X through in-depth analyses of three large spatial transcriptomic studies.

SPARK-X relies on a non-parametric covariance test for detecting spatial expression patterns. Its non-parametric feature distinguishes it from existing SE methods that are primarily parametric in nature. Non-parametric modeling in SPARK-X ensures its robust performance under various data generating processes in different spatial transcriptomic technologies, leading to calibrated *P* values and improved power for SE analysis. Such robust performance of SPARK-X is especially beneficial for analyzing spatial transcriptomic data that are both large-scale and sparse. Compared with small-sample studies, large-scale spatial transcriptomic studies are better powered, more reproducible, and are thus becoming increasingly common. We recognize, however, that many spatial transcriptomic studies are still carried out on moderate number of spatial locations and that some spatial transcriptomic data are non-sparse [11, 56]. Spatial transcriptomic data measured on a small number of spatial locations (e.g., hundreds or thousands) from early spatial transcriptomic technologies can often be modeled effectively through an over-dispersed Poisson distribution [6]. Consequently, parametric modeling of spatial count data through generalized linear mixed model framework such as the Poisson version of SPARK performs well for these technologies. Indeed, as demonstrated through our simulations, SPARK and SPARK-X have comparable power for detecting the hotspot pattern under small-sample settings, though SPARK-X outperforms SPARK on other patterns or with larger samples. The benefits of SPARK-X over SPARK on detecting SE genes can also be observed in other small data sets collected from other technologies. For example, on a human heart Visium data, which contains 20,904 genes measured on 4247 spots (Additional file 1: Figure S12), SPARK-X identified 1536 SE genes while SPARK identified 644 (533 overlapped; Additional file 1: Figure S12C). Both these methods produced calibrated *P* values under permuted null (Additional file 1: Figure S12A). On a human ovarian cancer Visium data, which contains 1198 genes measured on 3492 spots, SPARK-X identified 651 SE genes while SPARK identified 579 (474 overlapped; Additional file 1: Figure S13C). Importantly, many cancer-related KEGG and Reactome pathways can only be identified based on the SE genes detected by SPARK-X (Additional file 1: Figure S13G and Additional file 5: Table S7), highlighting the benefits of SPARK-X analysis. Besides small data, some recent large spatial transcriptomic technologies yield non-sparse count. For example, the STARmap technology measures expression levels on tens of thousands of spatial locations but only for two dozen of genes. Consequently, the read depth per gene from STARmap can be reasonably high, resulting in non-sparse high-count data with almost no zero values (Additional file 1: Table S1). For such high-count non-sparse data, the Gaussian distribution as employed in SPARK-G is an effective approximation to the underlying over-dispersed Poisson data generating process. We applied both SPARK-G and SPARK-X on the mouse visual cortex STARmap data that consists of 23 known cell type marker and 5 activity-regulated genes measured on 32,845 cells (Additional file 1: Figures S14 and S15). In the analysis, both SPARK-G and SPARK-X produced calibrated *P* values under permuted null and were able to detect all 28 genes as SE genes. Despite the similar performance of SPARK-G and SPARK-X for this non-sparse data, the memory saving by SPARK-X in this data remains substantial: while SPARK-G required 43.45 GB, SPARK-X only used 0.19 GB (Additional file 1: Table S1). Therefore, SPARK-X serves as an effective complement of existing SE analysis approaches, especially for large-scale spatial transcriptomic studies.

We have primarily used the projection covariance function to measure similarity in either gene expression or coordinates between location pairs. Such similarity measurement is expressed effectively as a product of two input variables from the location pair and quantifies their coordinated deviation from the mean. The product of two input variables is commonly used as a key ingredient in many covariance functions other than the projection covariance function [57–59]. While the product of two input variables represents one important similarity measurement, other similarity measurements exist. For example, one could use the Euclidean distance, minimum [60, 61], powered minimum [62], and other ways [63–65] to measure similarity between two variables. Different similarity measurements used in various covariance functions [64–66] can be easily incorporated into SPARK-X to achieve optimal detection of distinct spatial expression patterns. In the present study, we only used the projection covariance function as it allows us to achieve orders of magnitude of computational gains as compared with other approaches. The projection covariance function is also robust and well powered to detect a range of spatial expression patterns, both simple and complex, in simulations and real datasets. Despite these benefits of the projection covariance function, we note that the statistical power of the SPARK-X will likely benefit from the use of other covariance functions in addition to the projection covariance function. Due to computational reasons, we did not examine other covariance functions but instead explored the use of different transformations on the coordinates to incorporate into SPARK-X a wide range of distance covariance matrices. While the number of detected SE genes varies across different distance covariance matrices, combining the association evidence across all matrices as in SPARK-X achieves higher power than using any individual matrix alone (Additional file 1: Table S3). Future methodological development for exploring the use of other covariance functions as well as other transformations in a computationally efficient manner may help improve the power of SPARK-X further.

## Methods

### Method overview

We aim to identify genes that display spatial expression patterns, commonly referred to as SE genes, in large-scale spatially resolved transcriptomic studies. These large-scale studies rely on various high-throughput spatial transcriptomic technologies [13–15] and collect gene expression measurements on tens or hundreds of thousands of spatial locations. Gene expression measurements in these large-scale spatial transcriptomic studies are often in the form of low counts with a large fraction of zero values. For SE analysis, we examine one gene at a time and consider its expression measurements collected on *n* different spatial locations. We refer to the spatial locations as samples in the present study. Depending on the technology, a sample may be a single cell (in the case of STARmap technology) or a cell-sized local region (in the case of HDST technology) or a local region that consists of dozens of cells (in the case of Slide-seq and Visium technologies). The sampled locations have known spatial coordinates recorded during the experiment. We denote ***s***_***i***_ as the *d*-vector of spatial coordinates for *i*th sample, with *i* ∈ (1, …, *n*), and $\boldsymbol{S}={\left({\boldsymbol{s}}_1^T,\cdots, {\boldsymbol{s}}_n^T\right)}^T$ as the corresponding *n* × *d* matrix of spatial coordinates. These spatial coordinates vary continuously over a two-dimensional space (*d* = 2; ***s***_***i***_ = (*s*_*i*1_, *s*_*i*2_) ∈ *R*^2^) or a three-dimensional space (*d* = 3; ***s***_***i***_ = (*s*_*i*1_, *s*_*i*2_, *s*_*i*3_) ∈ *R*^3^) depending on the technology. We denote *y*_*i*_(***s***_***i***_) as the gene-expression measurement for the *i*th sample and ***y =*** (*y*_1_(***s***_1_), ***⋯***, *y*_*n*_(***s***_*n*_))^*T*^ as the *n*-vector of gene expression across all samples. We assume that both ***y*** and each coordinate of ***S*** has been centered and scaled to have mean 0 and standard deviation of 1. Centering and scaling do not influence type I error control but can affect statistical power. Here, our goal is to test whether the expression level of the gene of focus display any spatial expression pattern. Equivalently, we aim to test whether ***y*** is dependent on the spatial coordinates ***S***. We rely on a general class of covariance tests [24–27], which includes the Hilber-Schmidt independence criteria test [24] and the distance covariance test [25] as special cases, to perform SE analysis in a non-parametric fashion. Non-parametric testing ensures robust performance and wide applicability of our method to spatial transcriptomic data that are collected from various technologies with potentially different data features and different data generating mechanisms. Our method builds upon the following intuition: if ***y*** is independent of ***S***, then the spatial distance between two locations *i* and *j* would also be independent of the gene-expression difference between the two locations. Consequently, we can construct two sample by sample relationship matrices, one based on gene expression and one based on spatial coordinates, to examine whether these two matrices are more similar to each other than expected by chance alone.

Technically, we construct an expression covariance matrix based on the gene-expression levels as an *n* by *n* matrix ***E = y***(***y***^***T***^***y***)^***−*1**^***y***^***T***^. We also construct a distance covariance matrix for all samples based on spatial locations as an *n* by *n* matrix ***Σ = S***(***S***^***T***^***S***)^***−*1**^***S***^***T***^. We refer to both matrices as the covariance matrices as they are generated from the projection covariance function and possess the two key covariance matrix properties including being symmetric and positive semi-definite. A covariance matrix is also known as a kernel matrix and a covariance function is also known as a kernel function. The projection covariance function has been widely used in many applications in genetics [67–69]. For both matrices, we center them as ***E***_*C*_ ***= HEH*** and ***Σ***_*C*_ ***= HΣH***, where $\boldsymbol{H}=\left(\boldsymbol{I}-{\mathbf{1}}_n{\mathbf{1}}_n^{\boldsymbol{T}}/n\right)$ with ***I*** being an *n* by *n* identity matrix and **1**_*n*_ being an *n*-vector of 1s. Centering does not alter results here as we have already centered both ***y*** and ***S*** before constructing these covariance matrices. We then construct the following test statistic:

$$ T= trace\left({\boldsymbol{E}}_C{\boldsymbol{\varSigma}}_C\right)/n. $$

Intuitively, each element in either covariance matrix measures the similarity between pairs of locations in terms of their coordinated deviation from the mean. When *y* and ***S*** are independent of each other, the similarity measurement between a location pair in terms of gene expression will not be correlated with the similarity measurement between the location pair in terms of distance. Consequently, the test statistics *T*, which is effectively a summation of the products between the two similarity measurements across all location pairs, will be small. When *y* and ***S*** are not independent of each other, the similarity measurement in terms of gene expression will be correlated with the similarity measurement in terms of the distance across location pairs, thus leading to a large test statistics *T*. Formally, under the null hypothesis that ***y*** and ***S*** are independent of each other, *T* asymptotically follows a mixture of chi-square distributions [27]

$$ \frac{1}{n^2}{\sum}_{i,j}{\lambda}_{E,i}{\lambda}_{\varSigma, j}{z}_{ij}^2, $$

where *λ*_*E*, *i*_ is the *i*th ordered non-zero eigenvalue of ***E***_***C***_; *λ*_*Σ*, *j*_ is the *j*th ordered non-zero eigenvalue of ***Σ***_***C***_; and ${z}_{ij}^2$ are independent and identically distributed ${\chi}_1^2$ variables. An extremely large *T* that is rare under the above null distribution constitutes evidence against the null hypothesis. Consequently, we can compute a *P* value to measure the probability of encountering the same or a larger *T* as observed in the data based on the null distribution. The *P* value for testing the null hypothesis can be calculated using Davies’ exact method [70].

We employ several important algebraic manipulations to ensure that both computational complexity and memory requirement of our method are linear with respect to the number of spatial locations. First, we note that the eigenvalues of ***E***_***C***_ and ***Σ***_***C***_ are equivalent to the eigenvalues of (***y***^***T***^***y*** )^***−*1**^***y***^***T***^***Hy*** (a scalar) and (***S***^***T***^***S***)^***−*1**^***S***^***T***^***HS*** (a *d* × *d* matrix) [71], respectively. The computational cost for obtaining these eigenvalues based on the later forms is only *O*(*nd*^2^). Second, we note that

$$ Tr\left({\boldsymbol{E}}_{\boldsymbol{c}}{\boldsymbol{\Sigma}}_{\boldsymbol{c}}\right)= Tr\left(\boldsymbol{y}{\left({\boldsymbol{y}}^{\boldsymbol{T}}\boldsymbol{y}\right)}^{-\mathbf{1}}{\boldsymbol{y}}^{\boldsymbol{T}}\boldsymbol{H}\boldsymbol{\Sigma } \boldsymbol{H}\right)={\left({\boldsymbol{y}}^{\boldsymbol{T}}\boldsymbol{y}\right)}^{-\mathbf{1}} Tr\left({\boldsymbol{y}}^{\boldsymbol{T}}\boldsymbol{H}\boldsymbol{\Sigma } \boldsymbol{H}\boldsymbol{y}\right). $$

Consequently, we never need to compute ***E***, ***Σ*** and their centered versions ***E***_*C*_ and ***Σ***_*C*_ throughout the algorithm. Instead, we only need to compute the key quantities ***y***^***T***^***y***, ***y***^***T***^***Hy***, ***S***^***T***^***S***, ***S***^***T***^***HS***, and ***y***^***T***^***H*Σ*Hy***, all of which require at most *O*(*nd*^2^) computational complexity and *O*(*nd*) memory requirement. Specifically, these key quantities can be computed efficiently in the following forms:

$$ {\boldsymbol{y}}^{\boldsymbol{T}}\boldsymbol{Hy}={\left(\boldsymbol{Hy}\right)}^{\boldsymbol{T}}\left(\boldsymbol{Hy}\right)={\left(\boldsymbol{y}-\overline{y}\right)}^{\boldsymbol{T}}\left(\boldsymbol{y}-\overline{y}\right), $$

$$ {\boldsymbol{S}}^{\boldsymbol{T}}\boldsymbol{HS}={\left(\boldsymbol{HS}\right)}^{\boldsymbol{T}}\left(\boldsymbol{HS}\right)={\left(\boldsymbol{S}-\frac{\mathbf{1}{\mathbf{1}}^{\boldsymbol{T}}}{\boldsymbol{n}}\boldsymbol{S}\right)}^{\boldsymbol{T}}\left(\boldsymbol{S}-\frac{\mathbf{1}{\mathbf{1}}^{\boldsymbol{T}}}{\boldsymbol{n}}\boldsymbol{S}\right), $$

$$ {\boldsymbol{y}}^{\boldsymbol{T}}\boldsymbol{H}\boldsymbol{\Sigma } \boldsymbol{H}\boldsymbol{y}={\boldsymbol{y}}^{\boldsymbol{T}}\boldsymbol{H}\boldsymbol{S}{\left({\boldsymbol{S}}^{\boldsymbol{T}}\boldsymbol{S}\right)}^{-\mathbf{1}}{\boldsymbol{S}}^{\boldsymbol{T}}\boldsymbol{H}\boldsymbol{y}={\left(\boldsymbol{y}-\overline{y}\right)}^{\boldsymbol{T}}\boldsymbol{S}{\left({\boldsymbol{S}}^{\boldsymbol{T}}\boldsymbol{S}\right)}^{-\mathbf{1}}{\boldsymbol{S}}^{\boldsymbol{T}}\left(\boldsymbol{y}-\overline{y}\right). $$

Finally, we note that the quantities involving ***S***, including the computation of ***S***^***T***^***S*** and ***S***^***T***^***HS*** as well as the eigen decomposition of (***S***^***T***^***S***)^***−*1**^***S***^***T***^***HS***, only need to be performed once at the beginning and need not to be re-computed for every gene in turn. The quantities involving ***y***, including ***y***^***T***^***y***, ***y***^***T***^***Hy***, and ***y***^***T***^***H*Σ*Hy***, would vary across genes but could be computed efficiently relying on the sparsity of ***y***. Indeed, these three quantities can be computed with a computational complexity that scales linearly with respect to the number of samples with non-zero values, resulting in substantial computational savings for large sparse data. Therefore, our method has an overall computational complexity of *O*(*nd*^2^ + *pn* ′ *d*) and memory requirement of *O*(*nd*^2^), where *p* is the number of analyzed genes and *n*′ is the number of spatial locations with non-zero counts averaged across genes.

The statistical power of the above covariance test will inevitably depend on how the distance covariance matrix ***Σ*** is constructed and how it matches the true underlying spatial pattern displayed by the gene of interest. While the above projection kernel construction allows us to achieve orders of magnitude of computational gains as compared to other kernels such as the Gaussian and periodic kernels used in SPARK, it is likely not optimal in detecting every possible expression patterns encountered in real data. For example, the projection kernel is likely suboptimal in detecting focal expression patterns that are targeted by Gaussian kernels or periodical expression patterns that are targeted by periodic kernels. To ensure robust identification of SE genes across various possible spatial expression patterns, we consider different transformations of the spatial coordinates ***s***_***i***_ and subsequent construction of different distance covariance matrices. Specifically, we applied five Gaussian transformations on the coordinates ***s***_***i***_ = (*s*_*i*1_, *s*_*i*2_) to obtain five sets of transformed coordinates ***s***′_***i***_ = (*s*′_*i*1_, *s*′_*i*2_) , with ${s}_{i1}^{\prime }=\mathit{\exp}\left(\frac{-{s}_{i1}^2}{2{\sigma}_1^2}\right)$ being the transformed x-coordinate and ${s}_{i2}^{\prime }=\mathit{\exp}\left(\frac{-{s}_{i2}^2}{2{\sigma}_2^2}\right)$ being the transformed y-coordinate in each set. In the transformation, we used different smoothness parameters *σ*_1_ and *σ*_2_ in each set to cover a range of possible local covariance patterns. In addition, we applied five cosine transformations on ***s***_***i***_ to obtain another five sets of transformed coordinates ***s***′_***i***_, with ${s}_{i1}^{\prime }=\cos \left(\frac{2\pi {s}_{i1}}{\phi_1}\right)$ being the transformed x-coordinate and ${s}_{i2}^{\prime }=\cos \left(\frac{2\pi {s}_{i2}}{\phi_2}\right)$ being the transformed y-coordinate in each set. We also used different periodicity parameters *ϕ*_1_ and *ϕ*_2_ in each set to cover a range of possible periodic patterns. The transformation parameters *σ*_1_, *σ*_2_, *ϕ*_1_, and *ϕ*_2_ are predetermined using the 20%, 40%, 60%, 80%, and 100% quantiles of the absolute values of the *x* and *y* coordinates in the data. Using the empirical quantiles of the data to construct different covariance matrices follows the main ideas of [5, 6]. Compared to the alternative approach of fixing the transformation parameters to some predetermined values, using the quantiles of the data for transformation has the benefits of being invariant to any scale transformation of the original data and allows us to construct the distance covariance matrices in a data-dependent fashion.

We used each transformed ${\boldsymbol{s}}_{\boldsymbol{i}}^{\prime }$ to construct a distance covariance matrix as described above, resulting in a total of ten transformed distance covariance matrices in addition to the untransformed distance covariance matrix (Additional file 1: Figure S17). Intuitively, the kernel constructed based on the untransformed coordinates is likely useful to detect linear expression pattern across the coordinates. The kernels constructed based on the cosine transformed coordinates are likely useful to detect periodic expression patterns on the tissue. While the kernels constructed based on the Gaussian transformed coordinates are likely useful to detect focal expression patterns on the tissue. Therefore, combining the original and transformed distance covariance matrices would allow us to detect a wide variety of spatial expression patterns encountered in real data. To do so, we computed a *P* value using Davies’ method for each distance covariance matrix. We then combined all eleven *P* values into a single *P* value through the Cauchy *P* value combination rule [72, 73].

We have so far described the method in the absence of covariates. In the presence of covariates, we can replace the *n*-vector **1**_*n*_ in the ***H*** matrix with a corresponding covariate matrix ***X*** of dimensionality *n* by *q*. The covariate matrix contains a column of 1’s that represents the intercept, with the remaining columns representing the measurements for the *q*-1 covariates. Therefore, the centering matrix becomes ***H =*** (***I − X***(***X***^***T***^***X***)^***−*1**^***X***^***T***^). Despite this change, the other steps remain the same.

We refer to the above method as SPARK-X (SPARK-eXpedited), which is implemented in the SPARK R package with underlying efficient C/C++ code linked through Rcpp and with multiple threads computing capability. The software, together with all the analysis code for reproducing the results presented in the present study, are freely available at www.xzlab.org/software.html.

### Simulation designs

We performed extensive simulations to comprehensively evaluate the performance of SPARK-X along with several other existing methods. To make simulations as realistic as possible, we simulated data based on parameters inferred from two published data sets that include a spatial transcriptomic (ST) data set [11] and a Slide-seq data set (Additional file 1: Figure S16). The two data sets represent two different gene-expression data structures, with the ST data representing a moderately sparse data with 60% of zero values and the Slide-seq data representing a highly sparse data with 99.4% of zero values (Additional file 1: Table S1). In the simulations, we first randomly simulated the coordinates for a fixed number of spatial locations (*n*) through a random-point-pattern Poisson process. On these spatial locations, we simulated expression levels for 10,000 genes based on a negative binomial distribution with details provided below. These 10,000 genes were all non-SE genes in the null simulations and consisted of 1000 SE genes and 9000 non-SE genes in the power simulations. For both non-SE genes and SE genes, we varied the dispersion parameter of the negative binomial distribution to be either 0.1, 0.2, or 1 for the moderately sparse setting and to be either 1, 2.5, or 5 for the highly sparse setting. These values were selected to match the scale of dispersion parameter estimated in the two real data sets. For the non-SE genes, we varied the mean parameter of the negative binomial distribution to be either 0.005 or 0.5. The low value of 0.005 corresponds to the median mean estimate in the Slide-seq data and represents a highly sparse gene-expression setting. The high value of 0.5 corresponds to the median mean estimate in the ST data and represents a moderately sparse gene-expression setting. For the SE genes, we simulated their expression levels to display three distinct spatial patterns (hotspot, streak, and gradient patterns, Fig. 1D).

Specifically, for the first two spatial patterns, we created either a circle (for hotspot pattern) or a band (for streak pattern) in the middle of the panel and marked spatial locations residing in these areas. The size of the circle and the size of the band were designed so that the marked spatial locations inside these areas represent a fixed proportion of all spatial locations, with the proportion set to be either 10%, 20%, or 30%. The expression measurements of the non-marked spatial locations were randomly generated from a negative binomial distribution with the mean parameter set to be 0.005 or 0.5. For the moderately sparse setting, the expression measurements of the marked spatial locations were generated from a negative binomial distribution with a mean parameter being either 1.5, 2, or 3 times higher than that in the non-marked spatial locations (for 500 SE genes) or 2/3, 1/2, or 1/3 of that in the non-marked spatial locations (for 500 SE genes), representing low, moderate, or high SE signal strength, respectively. For the highly sparse setting, the expression measurements of the marked spatial locations were generated from a negative binomial distribution with a mean parameter being either 2, 3, or 4 higher than that in the non-marked spatial locations (for 500 SE genes) or 1/2, 1/3, or 1/4 of that in the in the non-marked spatial locations (for 500 SE genes), representing low, moderate, or high SE signal strength, respectively.

For the gradient pattern, the expression levels of a fraction of spatial locations (=20%, 30%, or 40%) were set either in an increasing order (for 500 SE genes) or a decreasing order (for the other 500 SE genes) along the x-axis. The three fractions used correspond to low, moderate, or high SE signal strength in this setting, respectively. In particular, we generated the expression measurements for all spatial locations from a negative binomial distribution. For each SE gene, we randomly selected a fraction of spatial locations where we assigned their gene-expression values in either increasing or decreasing order back to them based on their x-axis coordinates. In contrast, the expression measurements for the non-SE genes were randomly assigned to all spatial locations, regardless of their spatial locations.

In all these simulations, we varied the number of spatial locations (n = 300, 500, 1000, 2000, or 3000 for the moderate sparsity setting and n = 3000, 10,000, 20,000, 30,000, or 50,000 for the highly sparse setting), the expression sparsity level (moderate or high, as measured by the mean parameter in the negative binomial distribution), the noise level (low, moderate or high noise, as measured by the dispersion parameter in the negative binomial distribution), the SE strength (weak, moderate, or strong, as measured by fold change in the mean parameter for the first two spatial patterns and by the fraction of spatial locations displaying expression gradient for the third spatial pattern), as well as the fraction of spatial locations in the focal/streak area for the first two spatial patterns.

### Real data analysis

#### Slide-seq data

Slide-seq is a technology which enables transcriptome-wide measurements with 10-micron spatial resolution by transferring RNA from tissue sections onto a surface covered in DNA-barcoded beads with known positions and inferring the locations of RNA using a sequencing-by-ligation strategy. We obtained the Slide-seq dataset collected on the mouse cerebellum from Broad Institute’s single-cell repository (https://singlecell.broadinstitute.org/single_cell/) with ID SCP354. We used the file “Puck_180430_6” which contains 18,671 genes measured on 25,551 beads with known spatial location information. The bead size is approaching the size of mammalian cells (10 microns), though each bead may overlap with multiple cells. After filtering out mitochondrial genes and genes that are not expressed on any bead, we analyzed a final set of 17,729 genes on 25,551 beads. The data is highly sparse with 99.46% entries being 0 (Additional file 1: Table S1).

#### Slide-seqV2 data

Slide-seqV2 is a technology that builds upon on Slide-seq with modifications to library generation, bead synthesis, and array-indexing, thereby markedly improving the mRNA capture sensitivity. We obtained the Slide-seqV2 dataset collected on the mouse cerebellum from Broad Institute’s single-cell repository with ID SCP948. The data contains 23,096 genes measured on 39,496 beads with known spatial location information. The bead size is the same as that in the Slide-seq data. Following [36], we first cropped the region of interest and filtered out beads with UMIs less than 100. After filtering out mitochondrial genes and genes that are not expressed on any bead, we analyzed a final set of 20,117 genes on 11,626 beads. While the capture sensitivity is improved in the Slide-seqV2 as compared to Slide-seqV1, the Slide-seqV2 data is still highly sparse with 98.35% entries being zero (Additional file 1: Table S1).

We performed cell decomposition and conditional SE analysis on the Slide-seqV2 data. Specifically, we used the recently developed RCTD software [36] (v.1.0.0) to infer cell type composition on each spatial location. Following the original RCTD paper, we used a single-nucleus RNA-seq data [74] to serve as the reference panel for RCTD fitting, which contains 19 cell types. In the analysis, RCTD rejected 1494 beads and assigned cell type labels to 11,061 cells confidently from 9554 beads. We converted the inferred cell types into binary indicators and used them as covariates in SE analysis. Because RCTD produced confident cell type assignment using a set of 3338 genes (after RCTD filtering) on 11,061 cells (inferred from 9554 beads), we performed analysis on these genes and locations in the covariate adjusted SE analysis.

Besides conditional SE analysis, we also performed the cell type-specific SE analysis in the data. Specifically, we rely on the cell type composition estimates from RCTD to extract the locations that are dominated by Purkinje cells or granular cells. The Purkinje cells are primarily located in the thin Purkinje cell layer while the granule cells are primarily located in the thick granular layer; both layers are of highly irregular shapes. After removing genes with no expression on any of the selected cells, we performed SE analysis using SPARK-X for 3006 genes on 652 Purkinje cells and 3288 genes on 5891 granule cells.

#### High-definition spatial transcriptomics data

High-definition spatial transcriptomics is a method to capture RNA from tissue sections on a dense, spatially barcoded bead array, allowing transcriptome-wide measurements with 2-micron resolution. We obtained the HDST dataset collected on the mouse olfactory bulb from Broad Institute’s single-cell repository with ID SCP420. We used the file “CN24_D1” which contains 19,951 genes measured in 181,380 spots with known spatial location information. Each spot is a 2-micron well, approaching one fifth of the size of mammalian cells. We filtered out mitochondrial genes and genes that are not expressed on any spot and we removed spots with no gene-expression count. We analyzed a final set of 19,913 genes on 177,455 spots. The HDST data is extremely sparse with 99.96% of entries being 0 (Additional file 1: Table S1). We performed clustering analysis on the detected SE genes. To do so, for each gene in turn, we log transformed the raw count and scaled the transformed value further to have a mean of zero and standard deviation of one across all spots. We then used the hierarchical agglomerative clustering algorithm in the R package *amap* (v.0.8–18) to cluster identified SE genes into five gene groups.

We performed conditional SE analysis on a subset of the HDST data to examine the extent to which SE genes display spatial expression pattern beyond those explained by spatial distribution of cell types. To do so, we first extracted the most likely cell type for each spot based on the original publication and kept the spots with confident cell type assignment (*P*-adjust< 0.05). After filtering out mitochondrial genes and genes that are not expressed on any spots, we analyzed a final set of 17,121 genes on 103,602 spots. There are 63 cell types including non-neuronal cell types and multiple neuronal subtypes clustered in the original publication. We treated the assigned cell types as covariates for SE analysis on these spots.

We also performed cell type-specific SE analysis on a subtype of inhibitory neurons, the olfactory bulb inner horizontal cells (OBINH2). We selected these inhibitory neurons as they have the largest cell numbers among all cell types in the data. In the analysis, we extracted spatial locations that are labeled as OBINH2 neurons and removed genes that are not expressed on any of the extracted locations. In total, we analyzed 11,504 genes measured on 15,650 spatial locations in the cell type-specific SE analysis.

#### 10X Visium data

The 10X Visium is a platform that builds upon on the original Spatial Transcriptomics technology with improvements on both resolution (55-micron resolution, with smaller distance between barcoded regions) and experimental time. We obtained a Visium dataset collected on the human heart tissue from the 10X Visium spatial gene-expression repository (https://support.10xgenomics.com/spatial-gene-expression/datasets/1.1.0/V1_Human_Heart). The data contains 36,601 genes measured on 4247 spots with known spatial location information. Each spot is a 55-micron well. We filtered out mitochondrial genes and genes that are not expressed in any spot. We analyzed a final set of 20,904 genes on 4247 spots. The 10X Visium human heart data is relatively sparse with 90.81% of entries being 0 (Additional file 1: Table S1). In addition, we also obtained a Visium dataset collected on the human ovarian cancer tissue from the 10X Visium spatial gene-expression repository (https://support.10xgenomics.com/spatial-gene-expression/datasets/1.2.0/Targeted_Visium_Human_OvarianCancer_Pan_Cancer). The data contains 1198 genes measured on the 3493 spots. After removing spot with no gene-expression count, we analyzed a final set of 1198 genes on 3492 spots. Since this data is generated with an enriched library prepared using the Human Pan-Cancer Panel, only 73.78% of entries are 0.

#### STARmap data

Spatially resolved Transcript Amplicon Readout Mapping (STARmap) is a technology for 3D intact-tissue RNA sequencing. STARmap integrates hydrogel-tissue chemistry, targeted signal amplification, and in situ sequencing. We obtained the STARmap dataset collected on the mouse visual cortex from STARmap resources (https://www.starmapresources.com/data). We used the data collected in the sequentially encoded experiment, which contains 28 genes measured in 33,598 cells with known 3D spatial location information. These 28 genes include 23 cell type markers and 5 activity-regulated genes. After filtering out cells with no gene-expression count, we analyzed a final set of 28 genes on 32,845 cells. The STARmap data are of high counts with almost no zero values (1.2% zeros; Additional file 1: Table S1). We followed the procedures in the original paper [75] for cell type clustering. Specifically, we first applied log transformation to the raw count data and obtained the relative gene-expression levels through adjusting for the log-scale total read counts. We then clustered cells into inhibitory neurons, excitatory neurons, and non-neuronal cells using *Gad1*, *Slc17a7*, and four non-neuronal genes (*Flt1*, *Mbp*, *Ctss*, *Gja1*) using the K-means clustering algorithm.

For each of the above datasets, we performed permutations to construct an empirical null distribution of *P* values for each method by permuting the bead/spot/cell coordinates either ten times (for Slide-seq, Slide-seqV2, HDST, and 10X Visium data) or a thousand times (for STARmap data). Afterwards, we examined control of type I errors by the different methods on the basis of the empirical null distribution of *P* values. We declared an SE gene as significant based on an empirical FDR threshold of 0.01. We note that standard *P* value cutoffs such as Bonferroni-corrected *P* value threshold can also be used for SPARK-X due to its calibrated type I error control.

#### SE gene validation and functional gene set enrichment analysis

For the Slide-seq data, we obtained lists of genes that can be used to serve as unbiased validation for the SE genes identified by different methods. Specifically, we obtained the cerebellum gene list from the Harmonizome database [29], which consists of 2632 cerebellum-related genes identified in two datasets (Allen Brain Atlas adult mouse brain tissue gene-expression profiles; and TISSUES curated tissue protein expression evidence scores). In addition, we obtained a gene list from Wizeman et al. [30], which contains 4152 cell type markers genes in cerebellum. We used the two gene lists to validate the SE genes identified by different methods.

We also performed functional gene set enrichment analysis on the significant SE genes identified by SPARK-X and SPARK-G. We performed enrichment analyses using the R package clusterProfiler [76] (v.3.12.0) with GO terms and Reactome pathways. In the package, we used the default “BH” method for multiple-testing correction and set the default number of permutations to be 1000. We declared enrichment significance based on an FDR of 0.05.

#### Compared methods

We compared SPARK-X with three existing methods for detecting genes with spatial expression patterns: SPARK (v.1.1.0) [6], SPARK-G (the Gaussian version of SPARK), and SpatialDE (v.1.1.3) [5]. We did not include the trendsceek in the comparison due to its high computational burden. For example, it takes trendsceek 40 h to analyze a simulated data with 10,000 genes measured on 300 locations, which is 5000 times slower than SPARK-X. The computational burden of trendsceek becomes even heavier on larger datasets. We applied all four methods to the simulated data with SPARK being restricted to the settings that have a sample size ≤ 3000 due to its heavy computational burden. We applied SPARK-X, SPARK-G, and SpatialDE to the Slide-seq and Slide-seqV2 data. The SPARK-G and SpatialDE are not scalable when the sample size is over approximately 30,000. Therefore, we did not apply these two methods to the HDST data. The SpatialDE gave out error when we applied it to the STARmap data; thus, we only present the results from SPARK-X and SPARK-G there. For SPARK and SpatialDE, we adopted their default settings to filter data. Specifically, for SPARK, we filtered out genes that are expressed in less than 10% of the spatial locations and selected spatial locations with at least ten total read counts; for SpatialDE, we filtered out genes with aggregate expression count less than three and selected spatial locations with at least ten total read counts. For the SPARK-G, we did not perform any additional filtering. The number of analyzed gene for each method is provided in Additional file 1: Table S1.

## Supplementary Information

**Additional file 1: Supplementary information.** It includes all the supplementary figures and tables.

**Additional file 2: Supplementary Table 4.** Enrichment analysis of SE genes for the mouse cerebellum Slide-seq data.

**Additional file 3: Supplementary Table 5.** Enrichment analysis of SE genes for the mouse cerebellum Slide-seqV2 data.

**Additional file 4: Supplementary Table 6.** Enrichment analysis of SE genes for the mouse olfactory bulb HDST data.

**Additional file 5: Supplementary Table 7.** Enrichment analysis of SE genes for the human ovarian cancer Visium data.

**Additional file 6.** Review history.

## Data Availability

All codes, processed data, and analysis results in this paper are publicly available at GitHub [77] and Zenodo [78]. The source code is released under the MIT license. Slide-seq data, Slide-seqV2 data, and HDST data are available at Broad Institute’s single-cell repository (https://singlecell.broadinstitute.org/single_cell/) with ID SCP354, SCP948, and SCP420. The 10X Visium data sets are available at https://support.10xgenomics.com/spatial-gene-expression/datasets/1.1.0/V1_Human_Heart and https://support.10xgenomics.com/spatial-gene-expression/datasets/1.2.0/Targeted_Visium_Human_OvarianCancer_Pan_Cancer. The STARmap data set is available at https://www.starmapresources.com/data.
