# Supplementary material for: SPARK-X: non-parametric modeling enables scalable and robust detection of spatial expression patterns for large spatial transcriptomic studies
Source: Genome Biol. 2021 Jun 21;22:184. doi: 10.1186/s13059-021-02404-0 (PMC8218388; doi:10.1186/s13059-021-02404-0)
Supplement: Supplementary file 1 — Additional file 1: Supplementary information. It includes all the supplementary figures and tables. [file 13059_2021_2404_MOESM1_ESM.docx]

**Supplementary Figures**


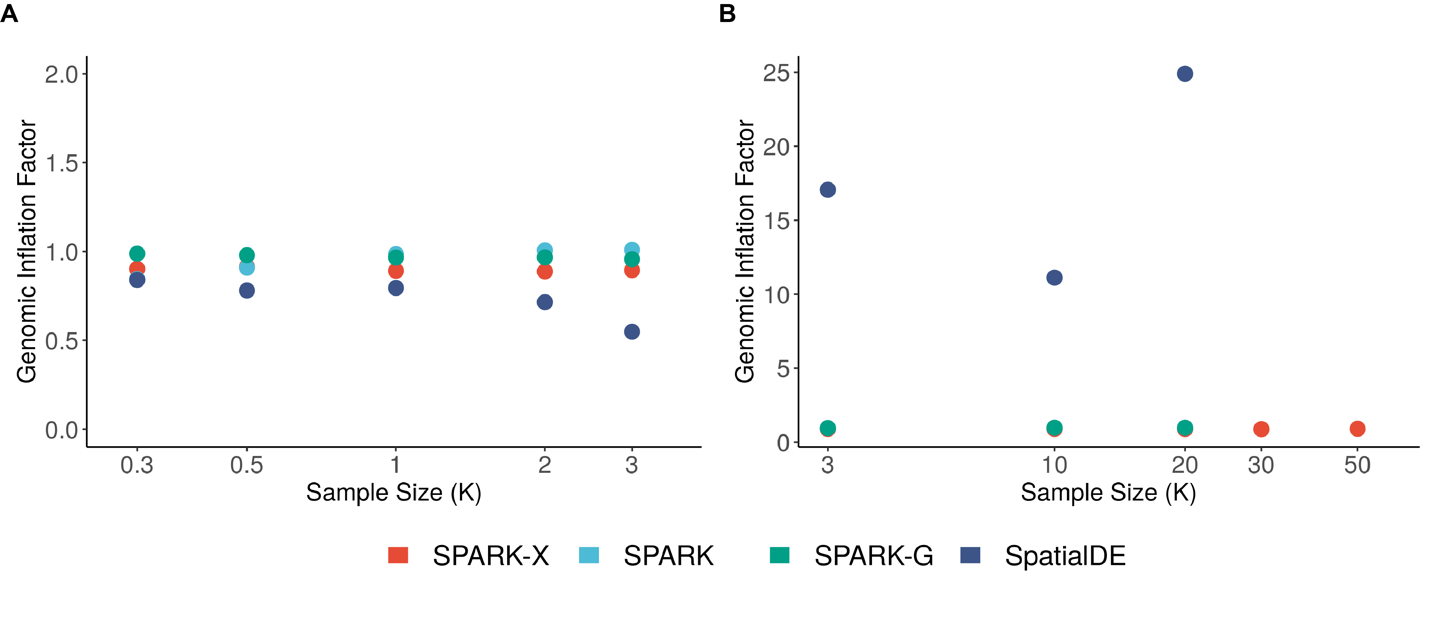
 **Fig. S1 Genomic inflation factor plot under the null condition for the simulations.** **(A)** Genomic inflation factor plot for null simulations with moderate sparsity ($\mu=0.5$) showing the genomic inflation factor across the sample size. Compared methods include SPARK-X (red), SPARK (sky blue), SPARK-G (green) and SpatialDE (steel blue). Simulations were performed under moderate dispersion (0.2). **(B)** Genomic inflation factor plot for null simulations with high sparsity ($\mu=0.005$) showing the genomic inflation factor across the sample size. Simulations were performed under moderate dispersion (2.5). Compared methods include SPARK-X (red), SPARK-G (green) and SpatialDE (steel blue).


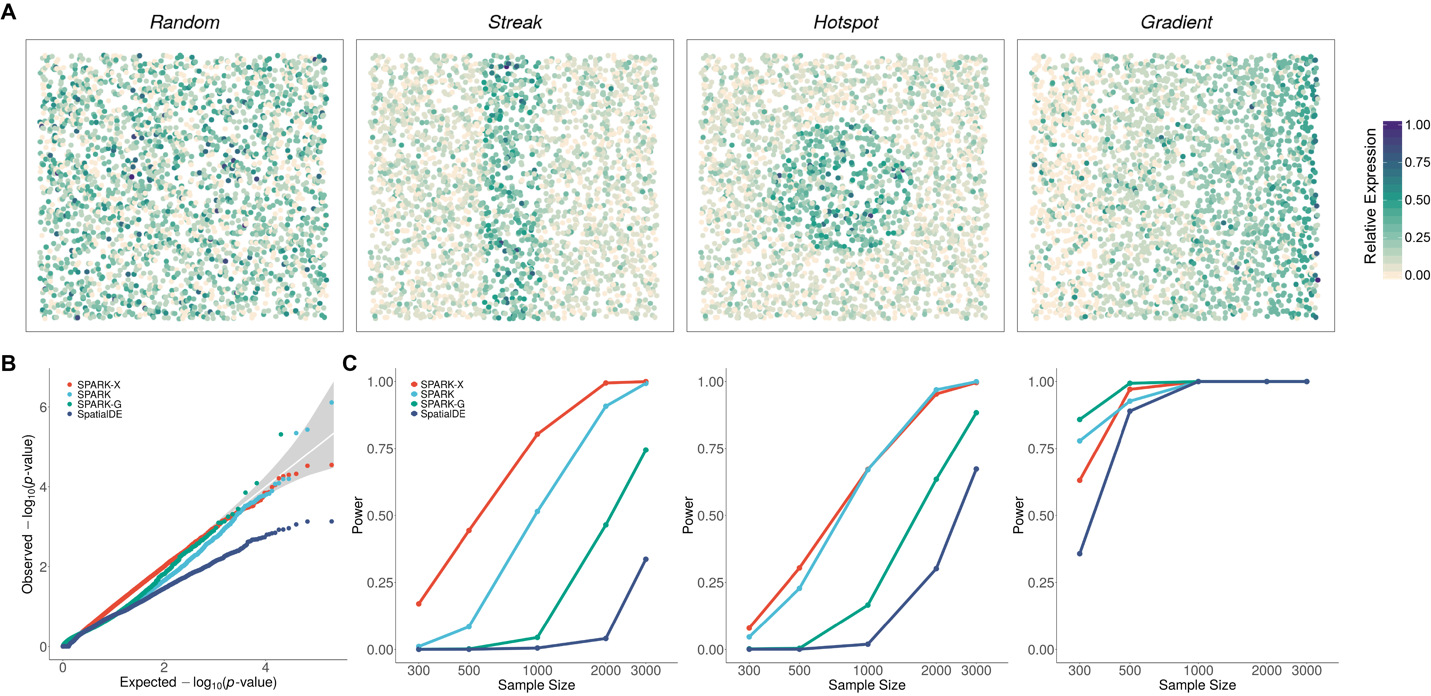


**Fig. S2 Comparison of different methods in the simulations with moderate sparsity under different sample sizes.** **(A)** Representative genes displaying random pattern and other three spatial expression patterns. Color represents relative gene-expression levels (green, high; antique-white, low). **(B)** Quantile–quantile plot of the observed −log_10_(*P*) from different methods against the expected −log_10_(*P*) under the null condition for the null simulations with moderate sparsity ($\mu=0.5$). *P* values were combined across ten simulation replicates. Simulations were performed under moderate sample size (n=1,000) and moderate dispersion (0.2). Compared methods include SPARK-X (red), SPARK (sky blue), SPARK-G (green) and SpatialDE (steel blue). **(C)** Power plots show the proportion of true positives (y axis) detected by different methods across a range of sample sizes (x axis) for alternative simulations with moderate sparsity at an FDR cutoff of 0.05. Simulations were performed under a moderate fraction of marked cells (20%) and moderate SE strength (twofold) for the hotspot and streak patterns or under moderate SE strength (30% cells displaying expression gradient) for the gradient pattern.


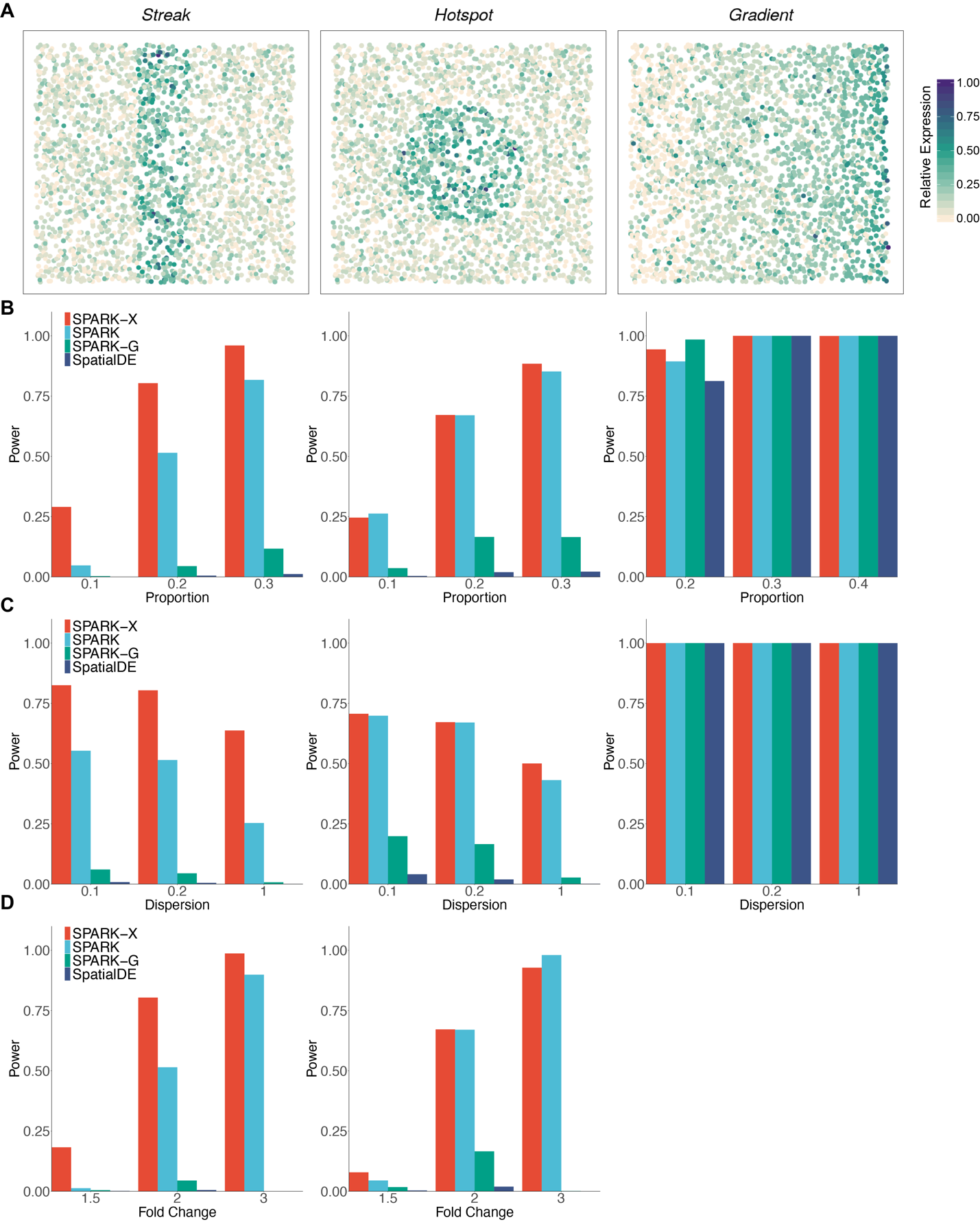


**Fig. S3 Power comparison of different methods in the alternative simulations with moderate sparsity. (A)** Representative genes displaying random pattern and other three spatial expression patterns. Color represents relative gene-expression levels (green, high; antique-white, low). **(B)** Power comparison of different methods in the alternative simulations under different fractions of marked cells. Simulations were performed under moderate SE strength (twofold), moderate sample size (n=1,000) and moderate dispersion (0.2). **(C)** Power comparison of different methods in the alternative simulations under different dispersions. Simulations were performed under moderate SE strength (twofold), moderate sample size (n=1,000) and moderate fraction of marked cells (0.2 for hotspot and streak, 0.3 for gradient). **(D)** Power comparison of different methods in the alternative simulations under different SE strengths. Simulations were performed under moderate sample size (n=1,000), moderate dispersion (0.2) and moderate fraction of marked cells (0.2). Compared methods include SPARK-X (red), SPARK (sky blue), SPARK-G (green) and SpatialDE (steel blue).


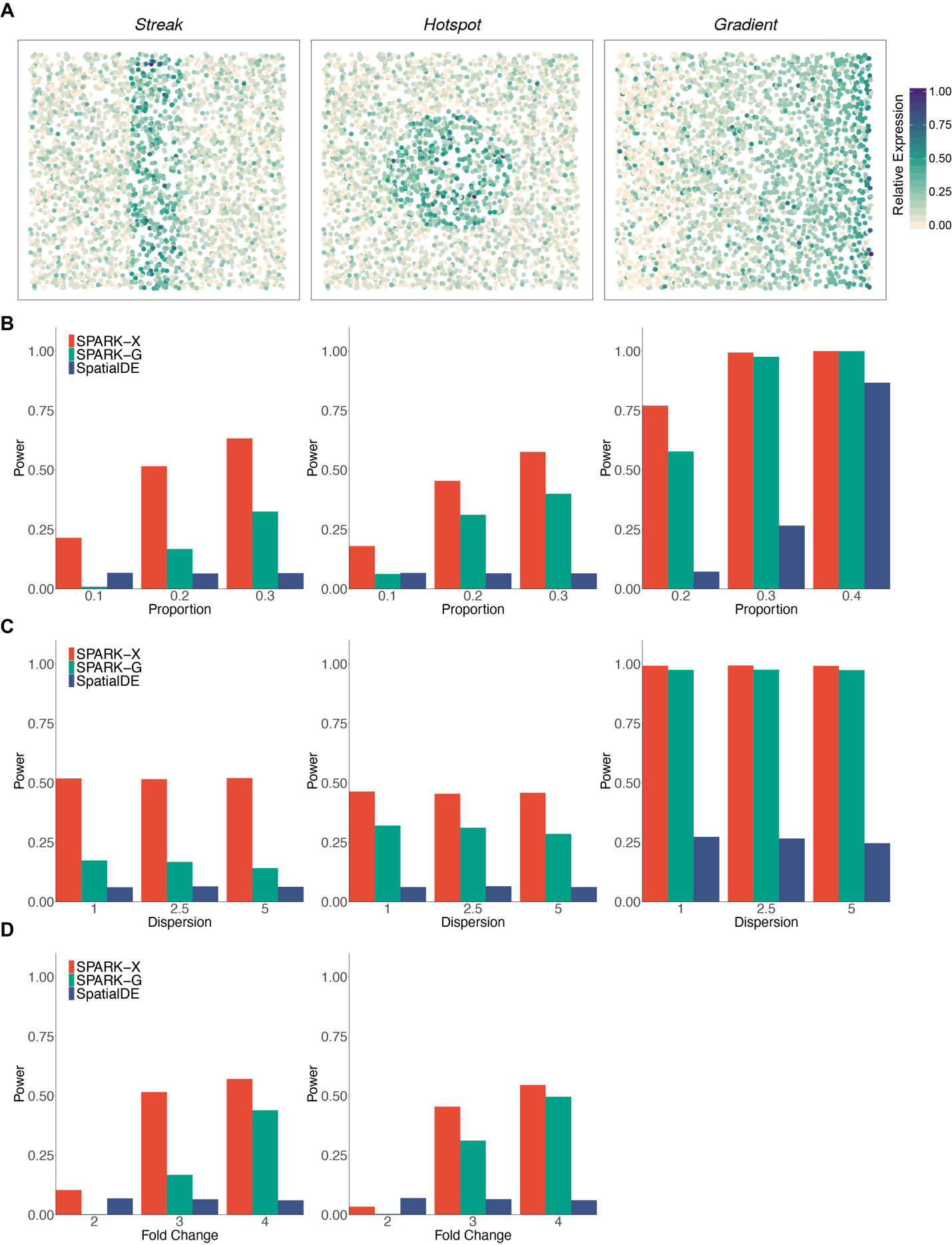


**Fig. S4 Power comparison of different methods in the alternative simulations with high sparsity. (A)** Representative genes displaying random pattern and other three spatial expression patterns. Color represents relative gene-expression levels (green, high; antique-white, low). **(B)** Power comparison of different methods in the alternative simulations under different fractions of marked cells. Simulations were performed under moderate SE strength (threefold), moderate sample size (n=20,000) and moderate dispersion (2.5). **(C)** Power comparison of different methods in the alternative simulations under different dispersions. Simulations were performed under moderate SE strength (threefold), moderate sample size (n=20,000) and moderate fraction of marked cells (0.2 for hotspot and streak, 0.3 for gradient). **(D)** Power comparison of different methods in the alternative simulations under different SE strengths. Simulations were performed under moderate sample size (n=20,000), moderate dispersion (2.5) and moderate fraction of marked cells (0.2). Compared methods include SPARK-X (red), SPARK-G (green) and SpatialDE (steel blue).


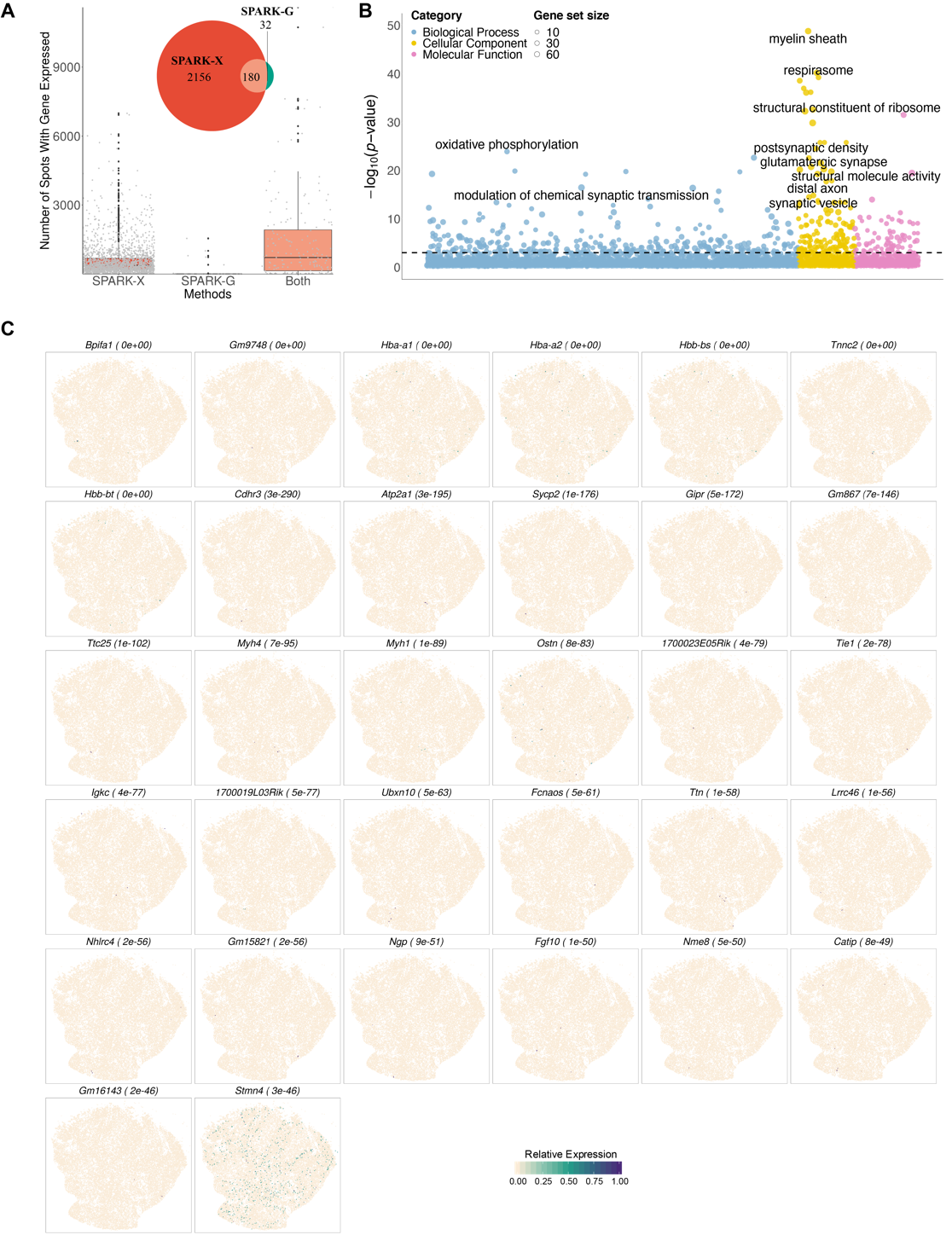


**Fig. S5 Analyzing the mouse cerebellum Slide-seq dataset (n = 25,551 beads**). **(A)** ﻿Boxplot displays the number of beads where significant SE genes are expressed and identified by different methods in the mouse cerebellum data. Results are shown for 2,156 genes that are detected by SPARK-X only (first column), 32 genes that are detected by SPARK-G only (middle column), and 180 genes that are detected by both methods (last column). Each grey dot represents a SE gene. ﻿A Venn diagram is embedded in the panel to show the overlap between SE genes identified by SPARK-X and SPARK-G. **(B)** Bubble plot of −log10(*P*) for pathway enrichment analysis on 2,336 SE genes obtained by SPARK-X in the Slide-seq data. The dashed line represents a *P* value cutoff of 0.05. Gene sets are colored by three categories: GO biological process (blue), GO molecular function (purple) and GO cellular component (yellow).  **(C)** Spatial expression pattern of 32 SE genes only identified by SPAKR-G. Color represents relative gene-expression levels (green, high; antique-white, low).


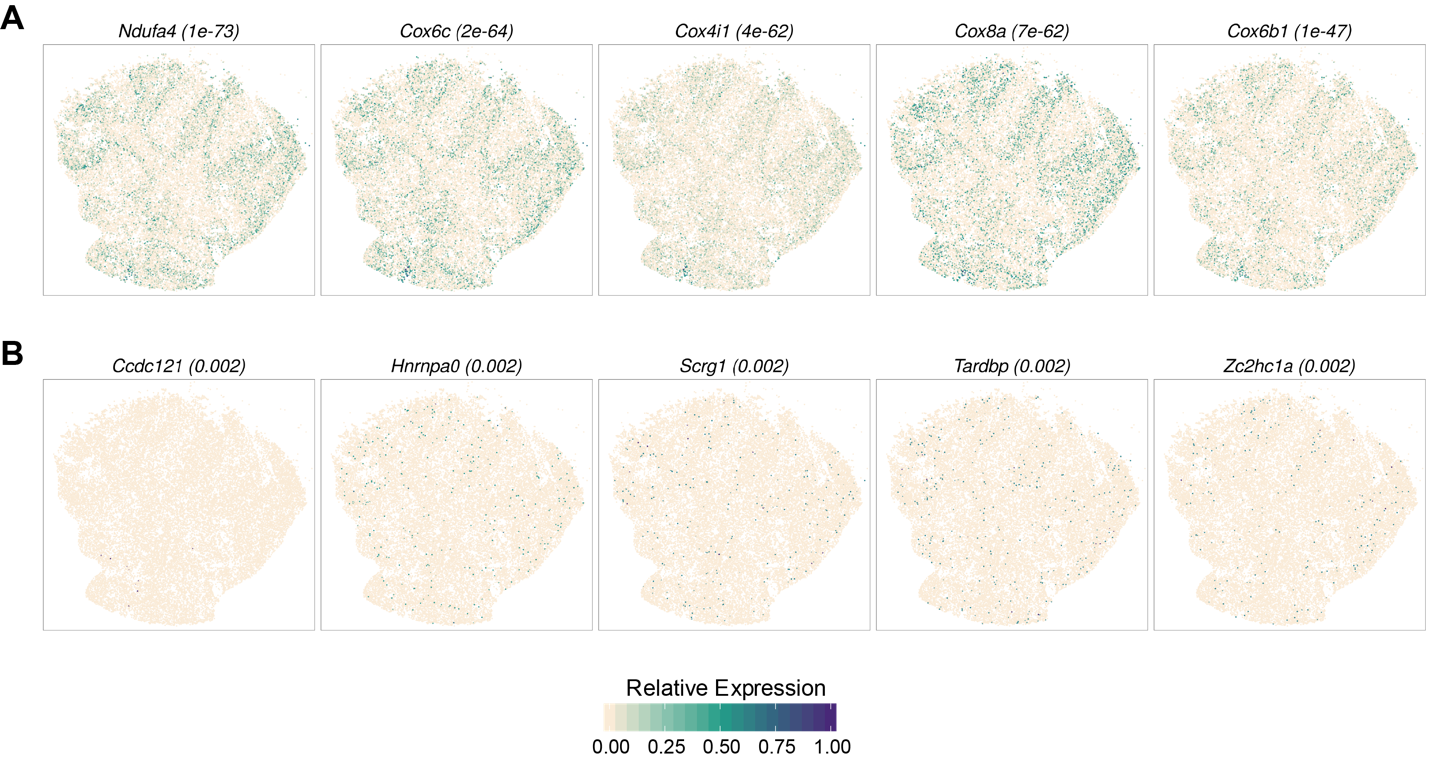


**Fig. S6 Spatial expression pattern of representative genes only identified by SPARK-X in the mouse cerebellum Slide-seq data.** SE genes are ranked from top to bottom based on *P*-values. **(A)** Spatial expression patterns of top five SE genes. **(B)** Spatial expression patterns of bottom five SE genes. Color represents relative gene-expression levels (green, high; antique-white, low). SPARK-X *P*-values are shown in parenthesis.


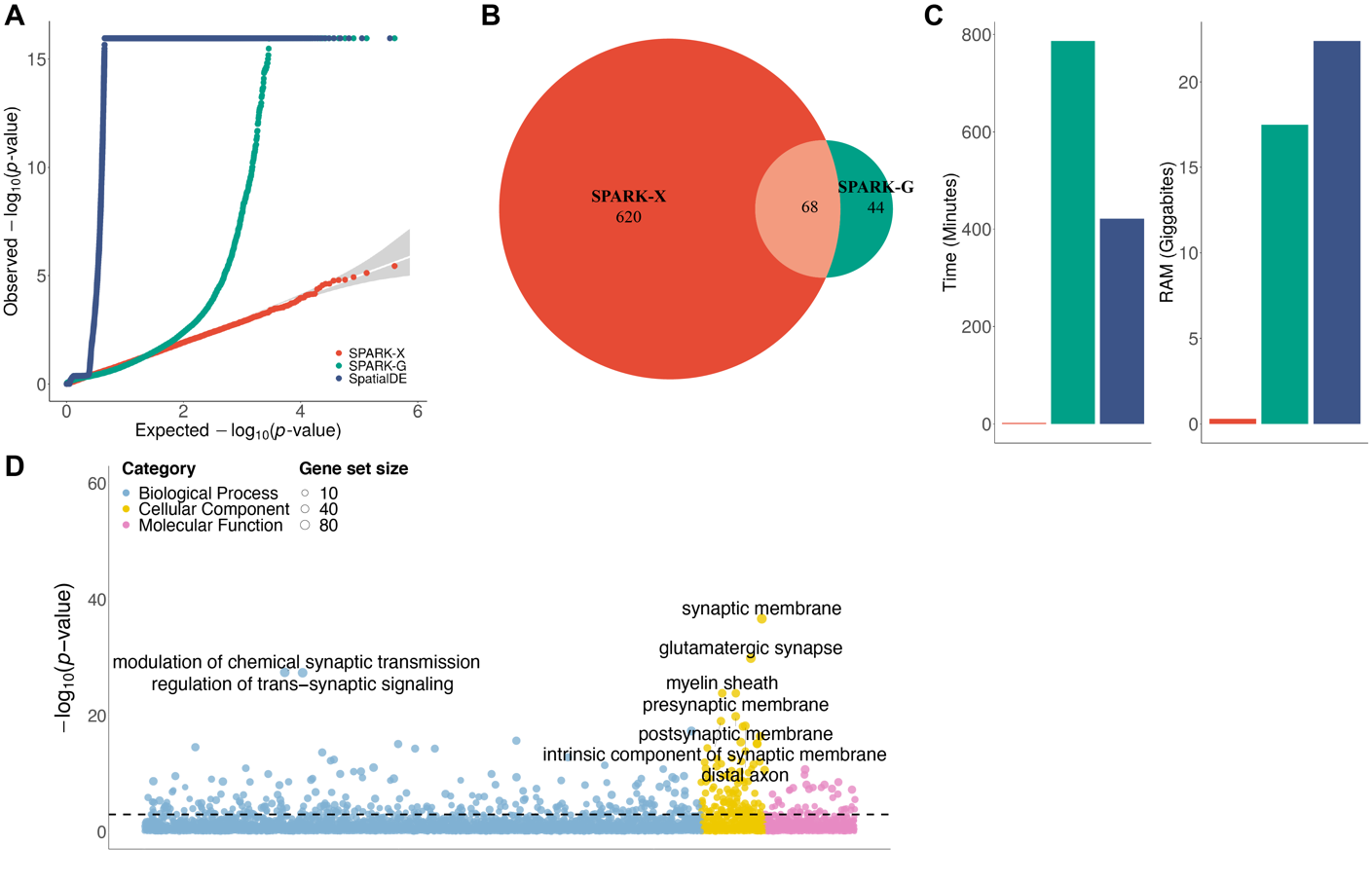


**Fig. S7 Analyzing the mouse cerebellum Slide-seqV2 dataset (n = 11,626 beads) .** **(A)** Quantile–quantile plot of the observed −log_10_(*P*) from different methods against the expected −log_10_(*P*) under the null condition in the permuted data. *P* values were combined across ten permutation replicates. Compared methods include SPARK-X (red), SPARK-G (green) and SpatialDE (steel blue). **(B)** ﻿ Venn diagram shows the overlap between SE genes identified by SPARK-X and SPARK-G. **(C)** Bar plots show the computation time and RAM of different methods for analyzing the mouse cerebellum Slide-seqV2 data. **(D)** Bubble plot of −log_10_(*P*) for pathway enrichment analysis on 688 SE genes obtained by SPARK-X. The dashed line represents a *P* value cutoff of 0.05. Gene sets are colored by three categories: GO biological process (blue), GO molecular function ﻿ (purple) and GO cellular component (yellow).


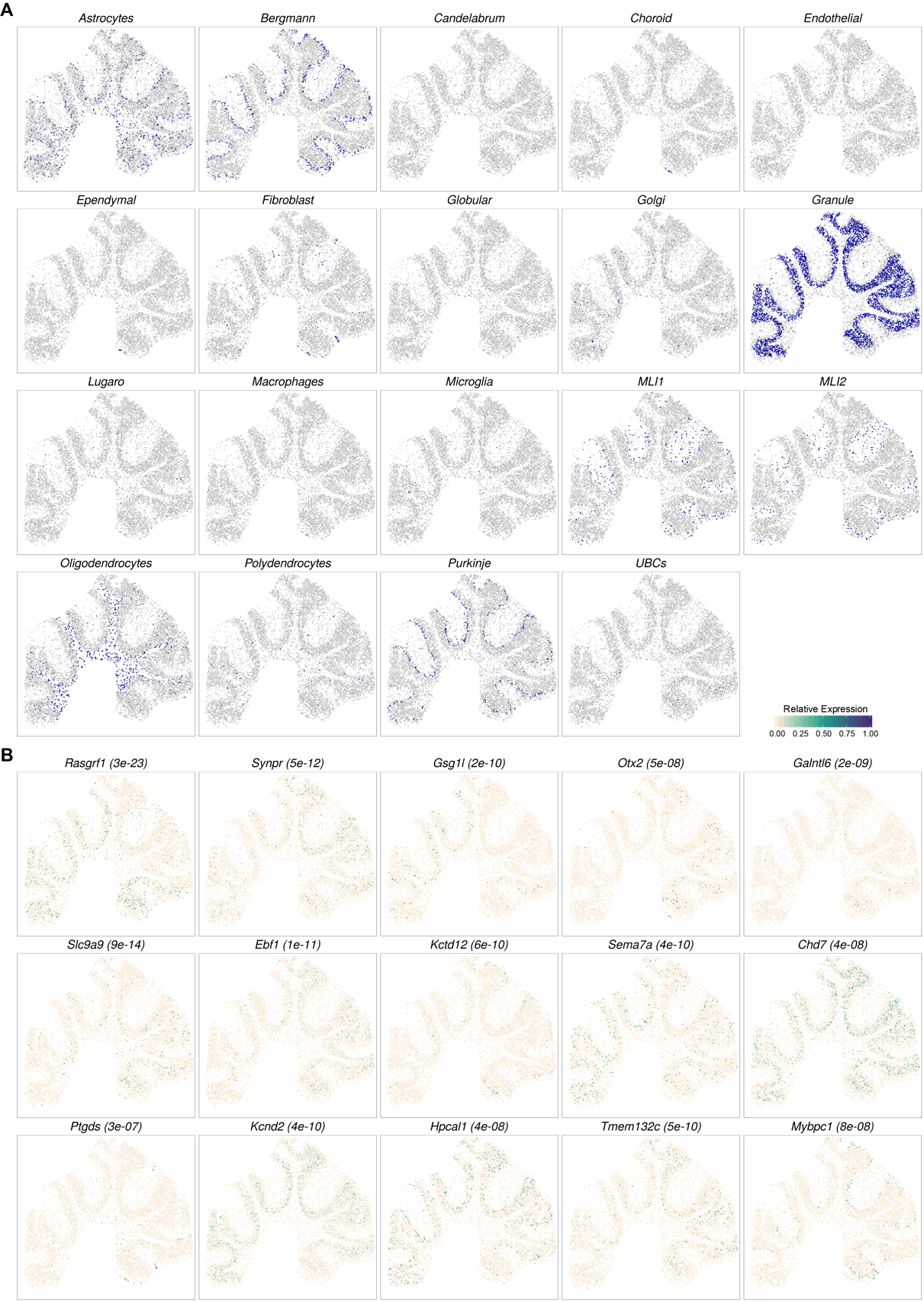


**Fig. S8 Cell type adjusted SE analysis in the mouse cerebellum Slide-seqV2 dataset (n = 11,061 cells). (A)** Predicted spatial localization of cell types by RCTD in mouse cerebellum Slide-seqV2 data. Purple color represents the predicted cell type while grey color represents background cell types. **(B)** Spatial expression pattern of the representative SE genes detected by SPARK-X after adjusting for cell types. Color represents relative gene-expression levels (green, high; antique-white, low). *P* values are shown inside paratheses.


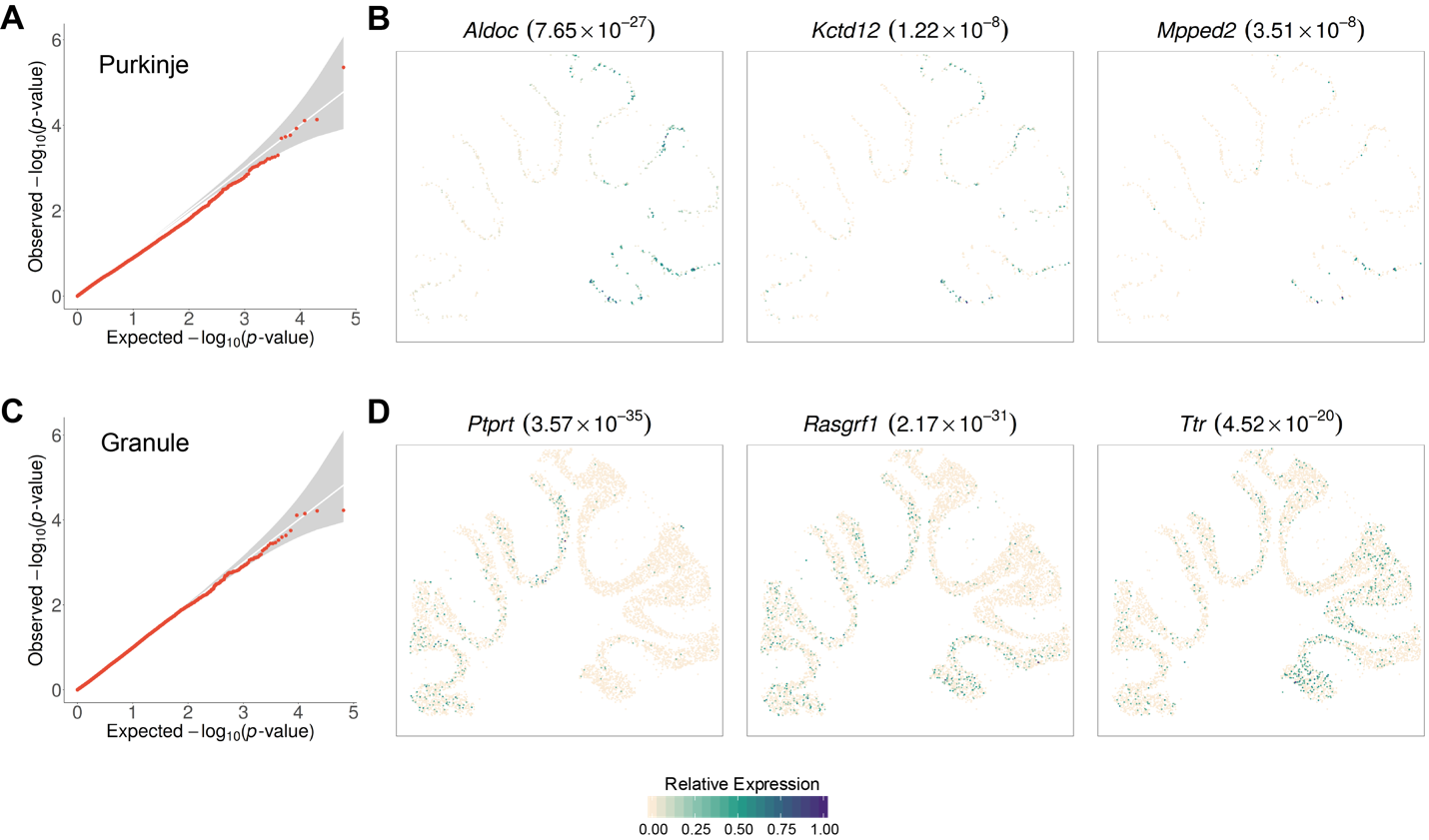


**Fig. S9** **Cell type specific SE analysis with SPARK-X.** Cell type specific analysis is performed on either Purkinje cells (**A-B**) or granule cells (**C-D**) in the Slide-seqV2 cerebellum data. (**A, C**). Quantile–quantile plot of the observed −log10(*P*) against the expected −log10(P) under the permuted null in the corresponding cell type. *P* values were combined across ten permutation replicates. Spatial expression pattern of representative SE genes detected by SPARK-X are shown for the Purkinje cell specific analysis (**B**) and granule cell specific analysis (**D**). In both B and D, the background antique white color represents cells that belong to the analyzed cell type.


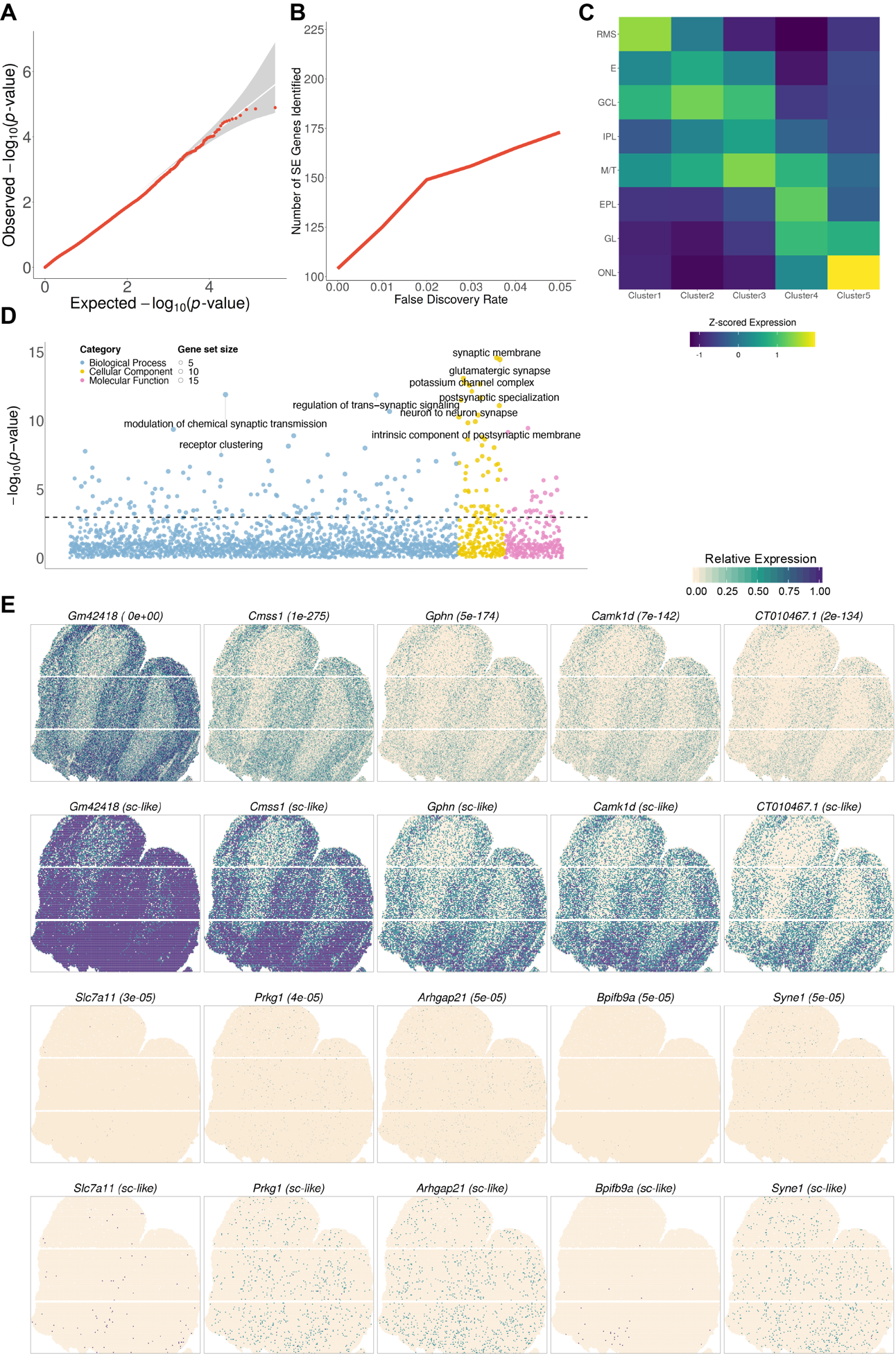


**Fig. S10 Analyzing the mouse olfactory bulb dataset (n = 177,455 spots**). **(A)** Quantile–quantile plot of the observed −log10(*P*) from SPARK-X against the expected −log10(*P*) under the null condition in the permuted data. *P* values in QQ plot were combined across ten permutation replicates. **(B)** Power plot shows the number of detected SE genes across a range of FDR. **(C)** Heatmap shows average expression of five gene clusters (columns) in eight major morphological layers (rows). Gene clusters are based on 125 SE genes and the average expression of each gene cluster is computed as the averaged Z-scored expression of all SE genes within the cluster. RMS, Rostral Migratory System; E, Ependymal Cell Zone; GCL, Granule Cell Layer; IPL, Internal Plexiform Layer; M/T, Mitral Layer; EPL, External Plexiform Layer; GL, Glomerular Layer; ONL, Olfactory Nerve Layer. **(D)** Bubble plot of −log10(*P*) for pathway enrichment analysis on 125 SE genes obtained by SPARK-X. The dashed line represents a *P* value cutoff of 0.05. Gene sets are colored by three categories: GO biological process (blue), GO molecular function (purple) and GO cellular component (yellow). **(E)** Spatial expression pattern of representative genes identified by SPARK-X in the mouse olfactory bulb HDST data. SE genes are ranked from top to bottom based on *P*-values. First row: spatial expression patterns of top five SE genes in the original resolution. Second row: spatial expression patterns of top five SE genes at the single cell level resolution. Third row: spatial expression patterns of bottom five SE genes in the original resolution. Fourth row: spatial expression patterns of bottom five SE genes at the single cell level resolution. Color represents relative gene-expression levels (green, high; antique-white, low). SPARK-X *P*-values are shown in parenthesis.


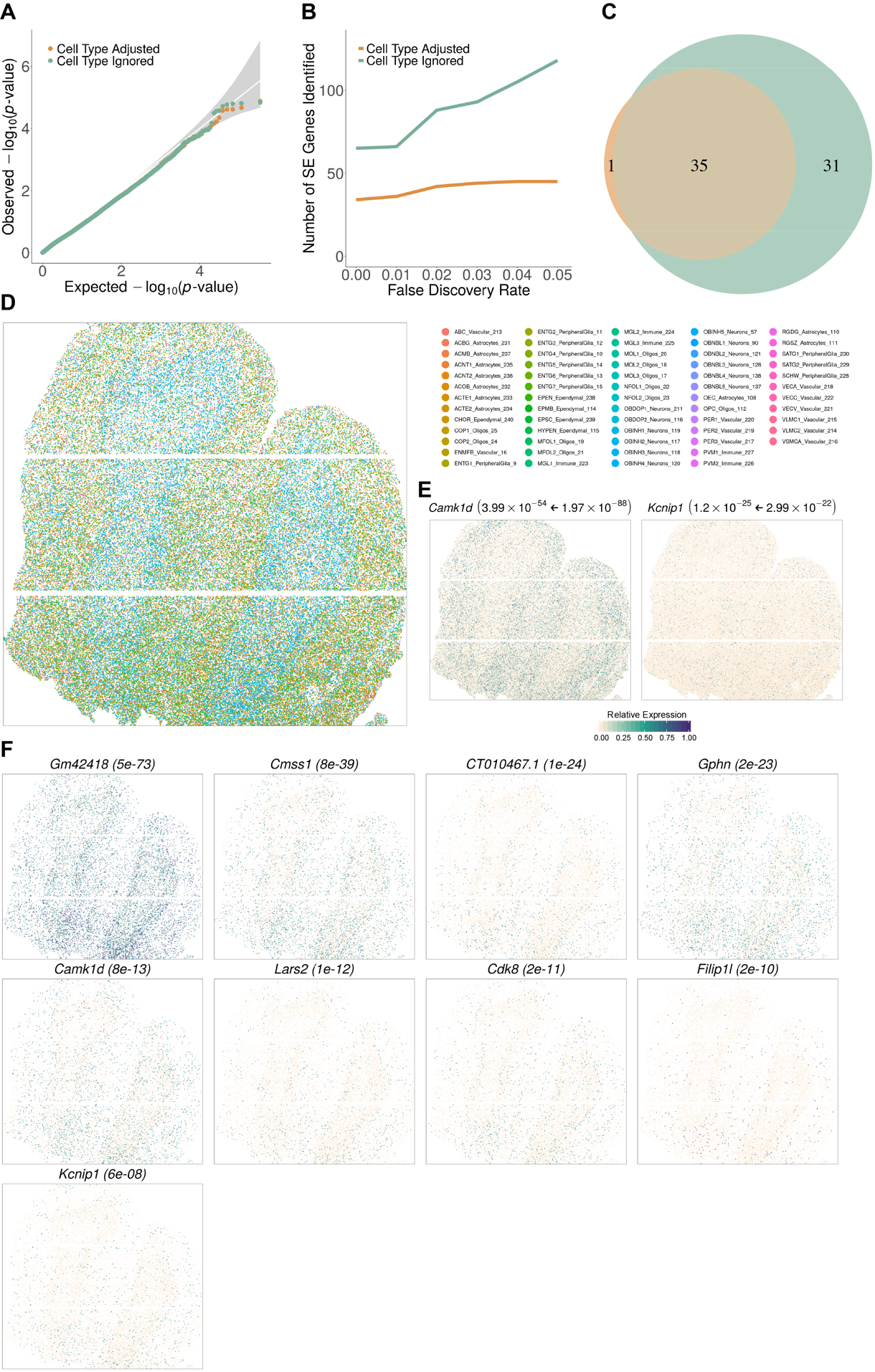


**Fig. S11 Conditional SE analysis (n = 103,602 spots**) **and cell type specific SE analysis** (**n = 15,650 spots**) **in** **the mouse olfactory bulb HDST dataset (A)** Quantile–quantile plot of the observed −log_10_(*P*) from SPARK-X against the expected −log_10_(*P*) under the null condition in the permuted HDST data. *P* values in QQ plot were combined across ten permutation replicates. Permutations were run with SPARK-X with (tan) or without (pale green) adjusting for cell types. **(B)** Power plot showing the number of detected SE genes across a range of FDR with (tan) or without (pale green) adjusting for cell types. **(C)** Venn diagram shows the overlap in SE genes identified by SPARK- X with (tan) or without (pale green) adjusting for cell types. **(D)** Cell type annotation in the HDST data. Spots are colored by cell types shown in the legend, where the cell type annotations were from ref.15. **(E)** Spatial expression patterns of two example SE genes at original resolution (green, high expression; antique-white, low expression). The background antique-white dots contain all 103,602 spots. *P* values from SPARK-X with (left side of the arrow) or without (right side of the arrow) adjusting for cell types are shown inside paratheses. **(F)** Spatial expression pattern of all nine SE genes identified by SPARK-X in the OBINH2 neurons. SE genes are ranked from top to bottom based on *P*-values. The background antique-white dots represent OBINH2 neurons (n = 15,650).


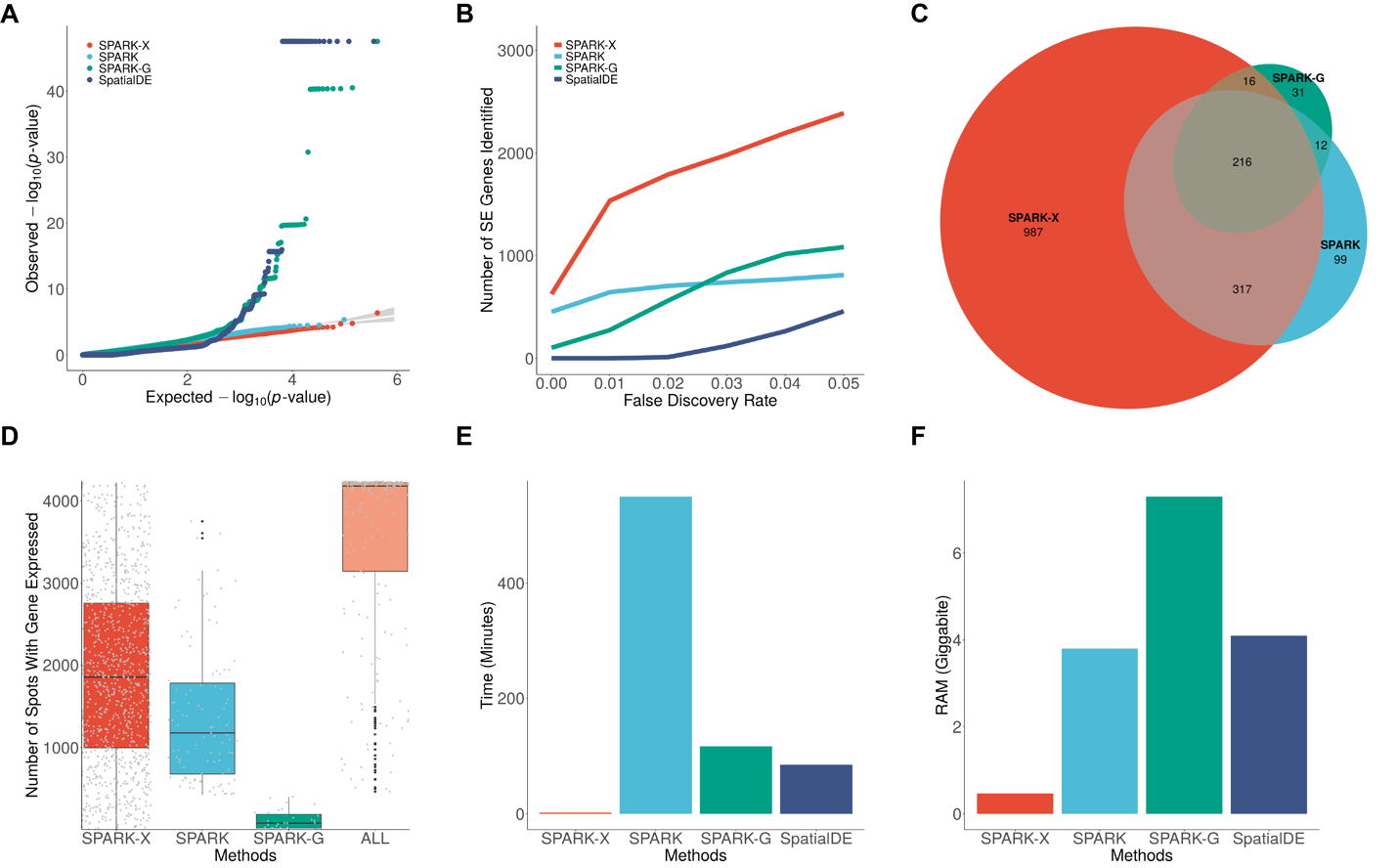


**Fig. S12 Analyzing the human heart Visium dataset (n = 4,247 spots**). **(A)** Quantile–quantile plot of the observed −log_10_(*P*) from SPARK-X against the expected −log_10_(*P*) under the null condition in the permuted data. *P* values were combined across ten permutation replicates. Compared methods include SPARK-X (red), SPARK (sky blue), SPARK-G (green) and SpatialDE (steel blue). **(B)** ﻿Power plot shows the number of genes with spatial expression pattern (y axis) identified by different methods at a range of FDRs (x axis) in human heart Visium data. **(C)** Venn diagram shows the overlap between SE genes identified by SPARK-X, SPARK and SPARK-G. **(D)** ﻿Boxplot displays the number of spots where significant genes are expressed and identified by different methods in the human heart Visium data. Results are shown for 987 genes that are detected by SPARK-X only (first column), 99 genes that are detected by SPARK only (second column), 31 genes that are detected by SPARK-G only (third column), and 216 genes that are detected by all methods (fourth column). Each grey dot represents a SE gene. ﻿**(E)** Bar plot shows the computation time of different methods for analyzing the human heart Visium data. SPARK analyzed 4,858 genes and SpatialDE analyzed 17,950 genes after their corresponding default filtering. The computation time here were adjusted assuming all methods analyzed all 20,904 genes to ensure a fair comparison. **(F)** Bar plot shows the RAM usage of different methods for analyzing the human heart Visium data.


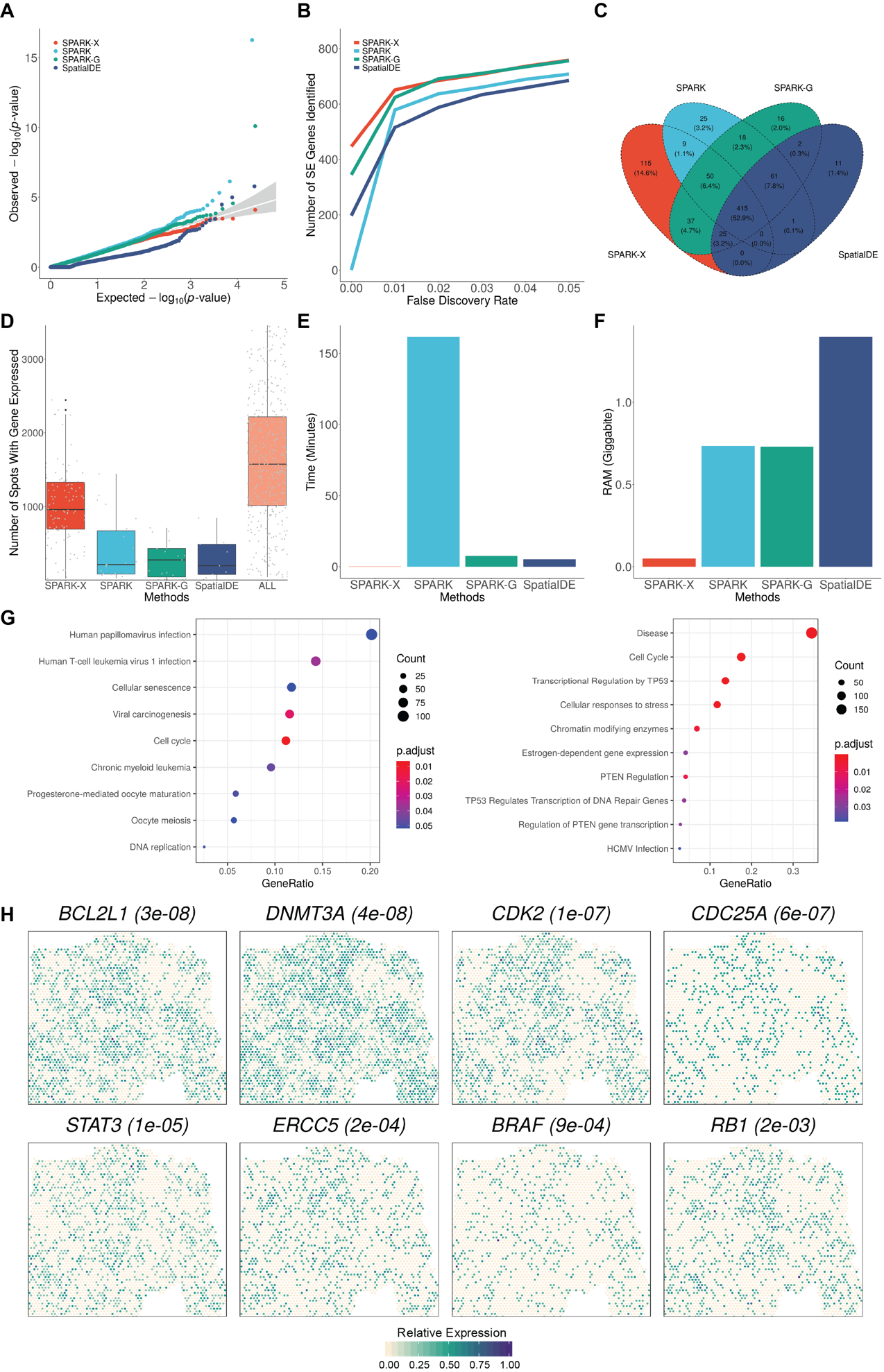


**Fig. S13 Analyzing the human ovarian cancer Visium dataset (n = 3,492 spots**). **(A)** Quantile–quantile plot of the observed −log_10_(*P*) from SPARK-X against the expected −log_10_(*P*) under the null condition in the permuted data. *P* values were combined across ten permutation replicates. Compared methods include SPARK-X (red), SPARK (sky blue), SPARK-G (green) and SpatialDE (steel blue). **(B)** ﻿Power plot shows the number of genes with spatial expression pattern (y axis) identified by different methods at a range of FDRs (x axis) in human ovarian cancer Visium data. **(C)** Venn diagram shows the overlap between SE genes identified by SPARK-X, SPARK, SPARK-G and SpatialDE. **(D)** ﻿Boxplot displays the number of spots where significant genes are expressed and identified by different methods in the human heart Visium data. Results are shown for 108 genes that are detected by SPARK-X only (first column), 22 genes that are detected by SPARK only (second column), 19 genes that are detected by SPARK-G only (third column), 9 genes that are detected by SpatialDE only (fourth column), and 414 genes that are detected by all methods (fifth column). Each grey dot represents a SE gene. ﻿**(E)** Bar plot shows the computation time of different methods for analyzing the human ovarian cancer Visium data. SPARK analyzed 1,025 genes and SpatialDE analyzed 1,158 genes after their corresponding default filtering. The computation time here were adjusted assuming all methods analyzed all 1,198 genes to ensure a fair comparison. **(F)** Bar plot shows the RAM usage of different methods for analyzing the human ovarian cancer Visium data. **(G)** Dot plots of enriched pathway (left: KEGG; right: Reactome) only presented using the SE genes identified by SPARK-X. **(H)** Spatial expression pattern for eight ovarian cancer related genes only detected by SPARK-X. Color represents relative gene-expression levels (green, high; antique-white, low).


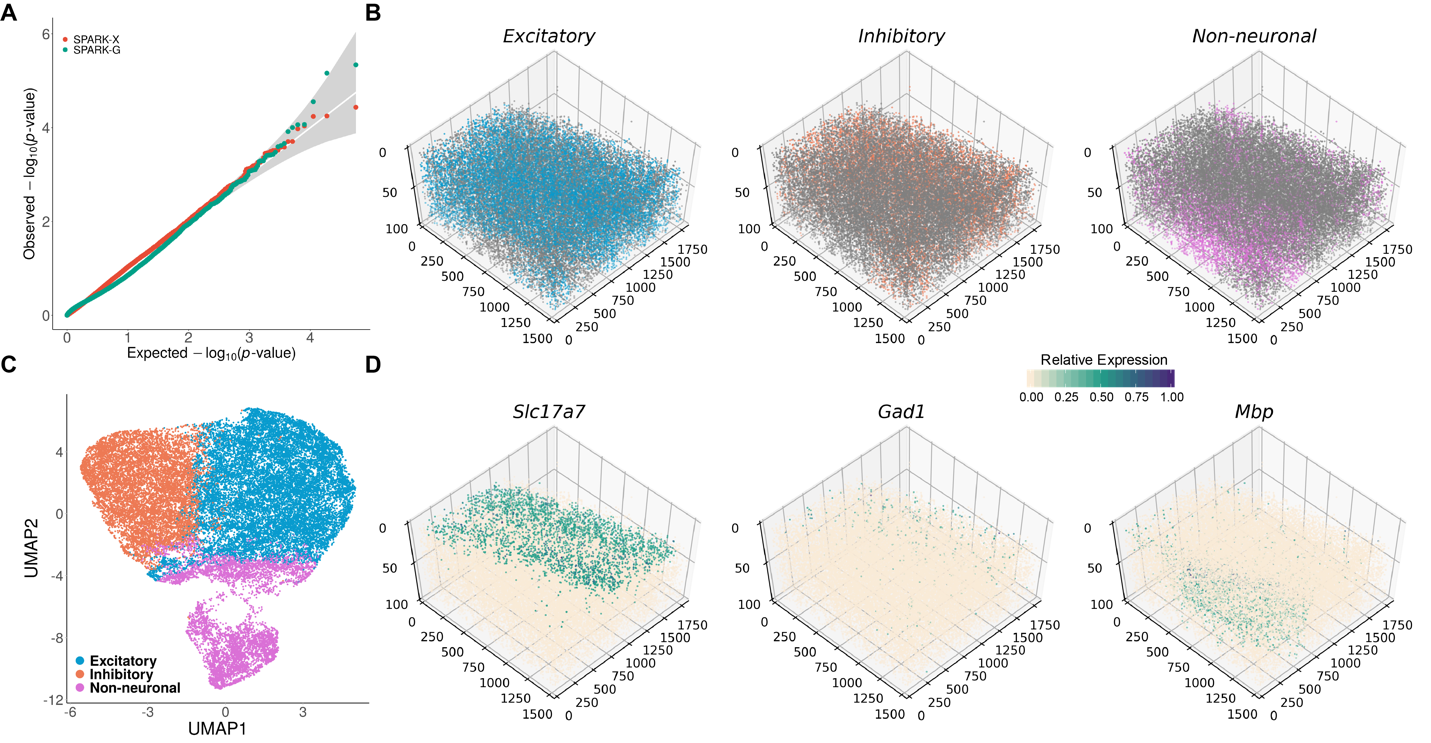


**Fig. S14 Analyzing the mouse visual cortex STARmap dataset (n = 32,845 cells**). **(A)** Quantile–quantile plot of the observed −log_10_(*P*) from SPARK-X against the expected −log_10_(*P*) under the null condition in the permuted data. *P* values were combined across a thousand permutation replicates. Compared methods include SPARK-X (red) and SPARK-G (green). **(B)** Spatial distribution of all three cell classes. The cell classes are represented by colored dots while all other background cells are shown as gray dots. The cell class information was obtained through K-means clustering. **(C)** UMAP visualization of 32,845 cells, with color representing three cell classes. **(D)** Spatial expression pattern for three representative genes. Color represents relative gene-expression levels (green, high; antique-white, low).


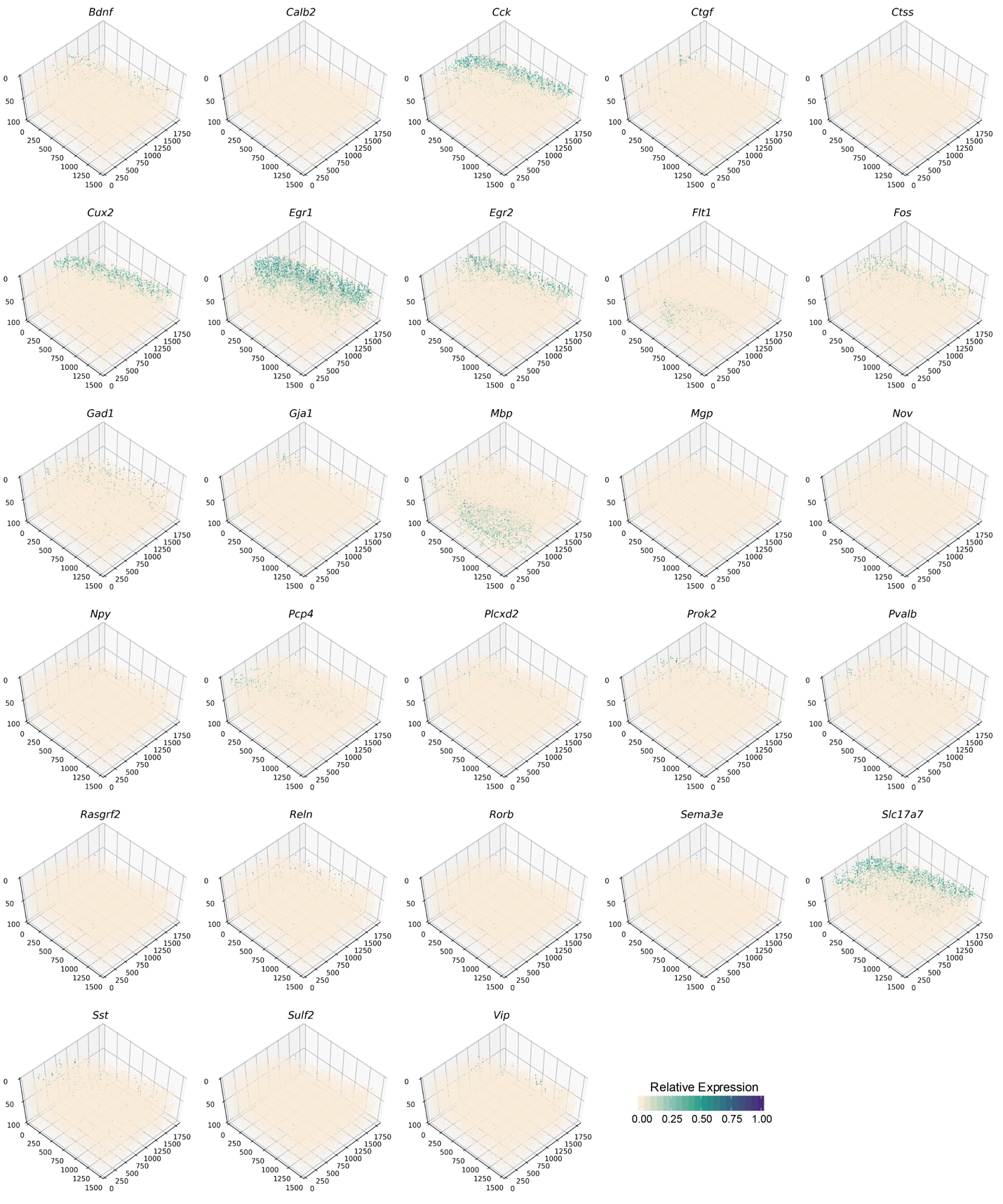


**Fig. S15 Spatial expression pattern of 28 SE genes identified by SPARK-X in the mouse visual cortex STARmap data.** Color represents relative gene-expression levels (green, high; antique-white, low).


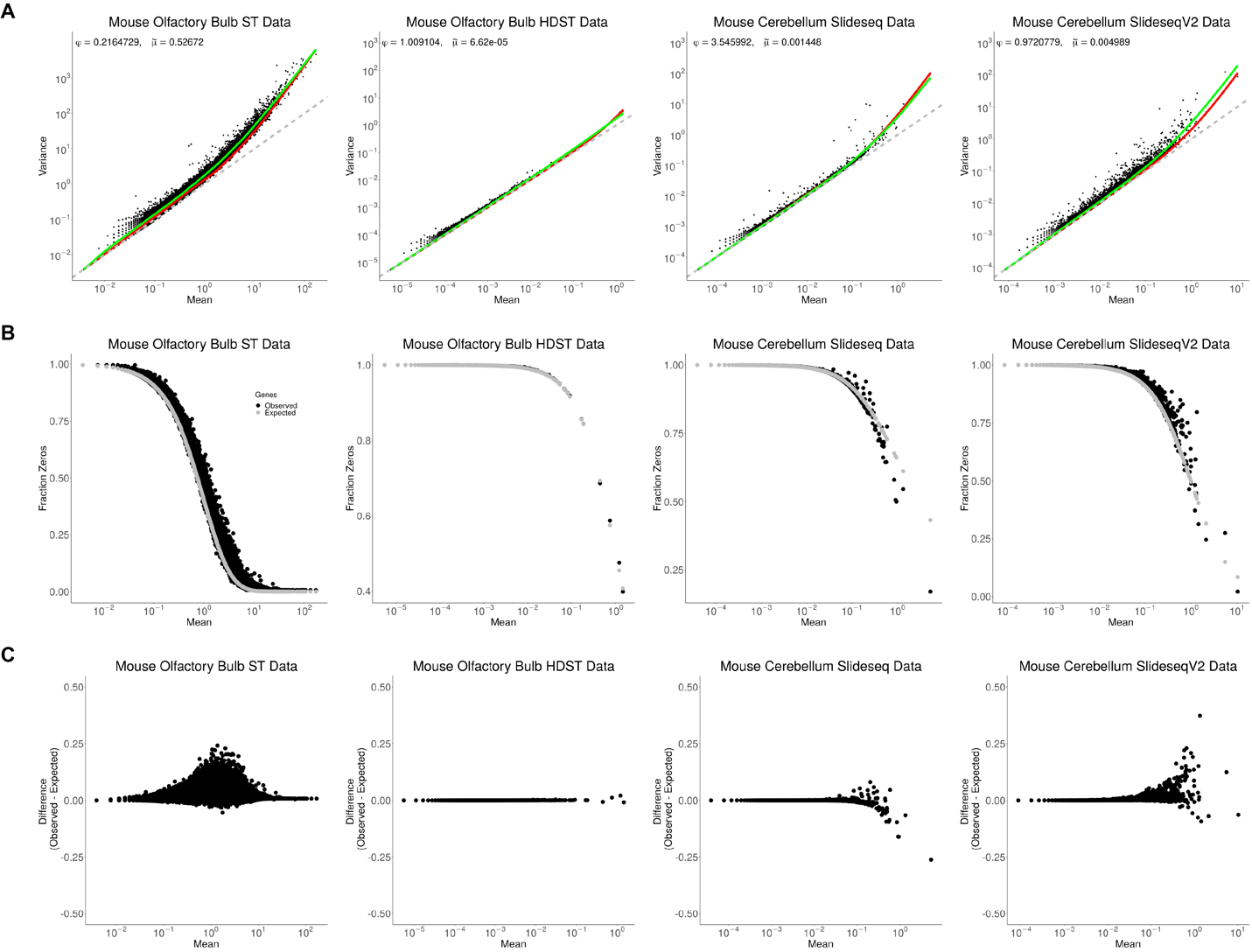


**Fig. S16** ﻿**Spatial transcriptomics data from different technologies can be captured reasonably well by over-dispersed Poisson distributions.** Four examined datasets are listed in columns including the mouse olfactory bulb ST data, the mouse olfactory bulb HDST data, the mouse cerebellum Slide-seq data, and the mouse cerebellum Slide-seqV2 data. **(A)** Mean-variance plots. Each dot represents a gene, whose mean (x- axis) and variance (y-axis) were calculated. A loess curve (green) and a quadratic function (red) is fitted for each dataset, where a dashed grey line shows the diagonal where the variance equals the mean. **(B)** Scatter plots show the fraction of zero counts of each gene versus the mean count of the gene. Each dot represents a gene. In each panel, the grey smooth curve shows the expected fraction of zeros under an over-dispersed Poisson model with a common dispersion parameter estimated across genes. **(C)** The difference between the observed and expected fractions under the over-dispersed Poisson model (y axis) for each gene is displayed against its mean count (x axis). Each dot represents a gene.


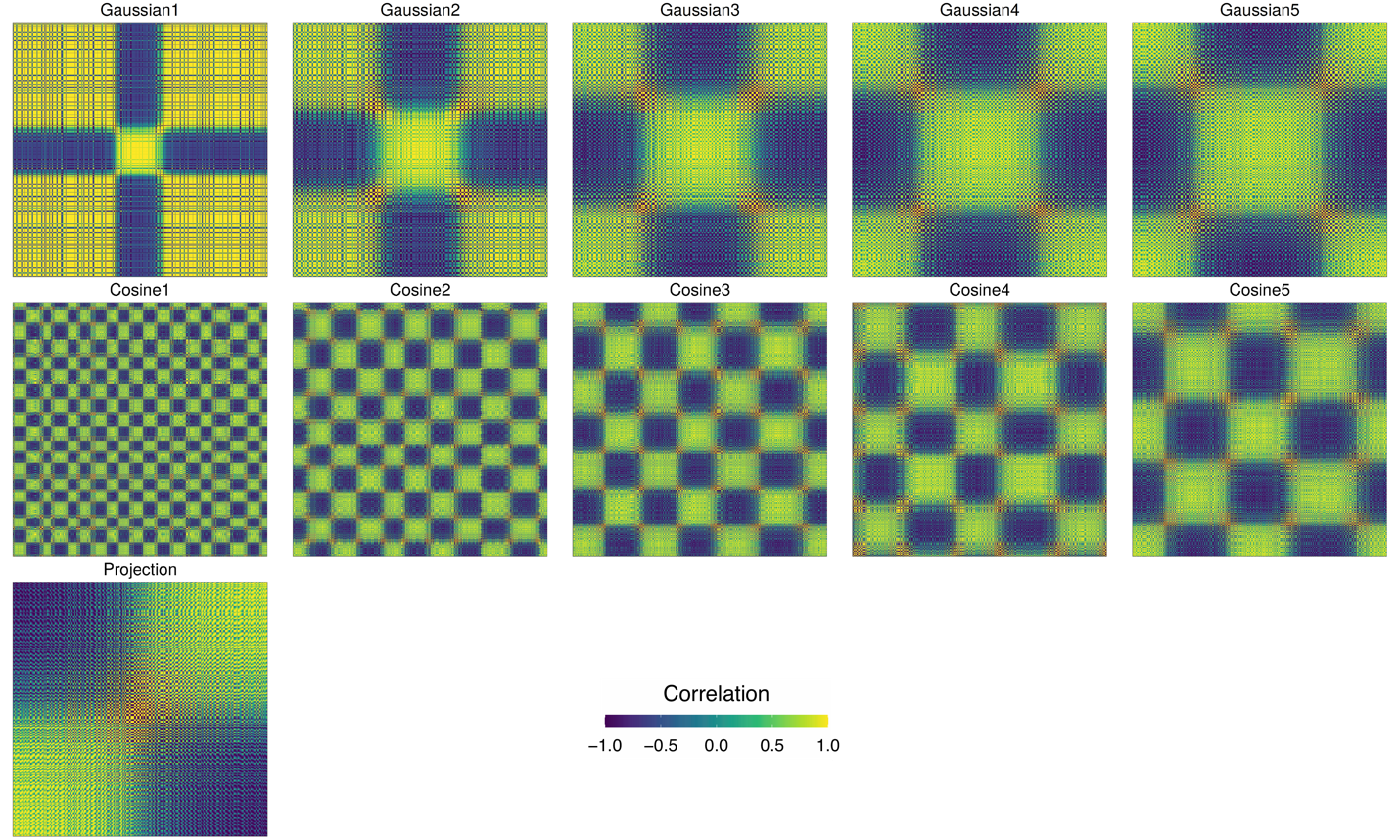


**Fig. S17** ﻿**Heatmaps illustrates eleven spatial kernels incorporated in SPARK-X.** Eleven kernels include five Gaussian transformed projection kernels, five cosine transformed projection kernels, and one direct projection kernel.

﻿**Table S1: Computation time in minutes and memory cost in gigabytes for analyzing the five real datasets using different methods**. Computations are carried out using a single thread of an Intel Xeon E5-2683 2.00GHz processor.

| **Data Set** | | **Mouse Cerebellum (SlideseqV1)** | **Mouse Cerebellum (SlideseqV2)** | **Mouse**  **Olfactory Bulb**  **(HDST)** | **Mouse**  **Visual Cortex (STARmap)** | **Human Heart (Visium)** | **Human Ovarian Cancer**  **(Visium)** |
| --- | --- | --- | --- | --- | --- | --- | --- |
| **Number of Samples** | | 25,551 | 11,626 | 177,455 | 33,598 | 4,247 | 3,492 |
| **Number of Genes** | | 17,729 | 20,117 | 19,913 | 28 | 20,904 | 1,198 |
| **Median Number of Reads Per Gene** | | 37 | 58 | 12 | 1.4E+08 | 94 | 810 |
| **Median Percentage of Zero Count Per Gene** | | 99.86% | 99.54% | 99.99% | 0.01% | 97.86% | 80.74% |
| **Overall Zero Count Percentage** | | 99.46% | 98.35% | 99.96% | 1.2% | 90.81% | 73.78% |
| **Number of Genes Analyzed (SpatialDE)** | | 14,794 | 16,722 | — | NA** | 17,950 | 1,158 |
| **Computational Time (Minutes)** | **SPARK-X** | 1.76 | 1.97 | 2.76 | 0.08 | 1.93 | 0.04 |
|  | **SPARK-G** | 3,362 | 781 | 114 (d)* | 27 | 117 | 8 |
|  | **SpatialDE** | 2,809^#^ | 509^#^ | 80 (d)* | NA** | 85^#^ | 5 |
| **Memory Cost (Gigabytes)** | **SPARK-X** | 0.28 | 0.33 | 0.42 | 0.19 | 0.47 | 0.1 |
|  | **SPARK-G** | 43.5 | 17.5 | 2,100* | 43.45 | 7.3 | 0.73 |
|  | **SpatialDE** | 71.7 | 22.4 | 3,500* | NA** | 4.1 | 1.40 |

﻿*: Estimated time and memory cost. We didn’t apply SpatialDE and SPARK-G to analyze the full HDST data due to the high computation requirements.

﻿**: SpatialDE gave out error when applied to this data and cannot analyze it.

^#^: Computation time are for analyzing all genes to ensure a fair comparison. Different methods employ different filtering criteria and analyze a different number of genes in different datasets in practice.

**Table S2: Percentage of failures in SPARK and the negative binomial model in simulations with different data sparsity levels.** The negative binomial model is implemented in glm.nb in the *MASS* R package. We set maxit to be 500 in glm.nb. The failure percentage was calculated based on the number of failures in 1,000 simulation replicates. In the simulation, we varied the mean parameter of the negative binomial distribution (0.005, 0.01,0.02,0.03,0.04,0.05,0.1,0.2) to generate data with different sparsity levels. The sample size is set to be 3000 and the dispersion parameter is set to be 0.2.

| **Zero%** | **99.5%** | **99%** | **98%** | **97%** | **96%** | **95%** | **90%** | **80%** |
| --- | --- | --- | --- | --- | --- | --- | --- | --- |
| **SPARK** | 97.5% | 52.9% | 0.8% | 0.1% | 0% | 0% | 0% | 0% |
| **GLM.NB** | 75.2% | 59.1% | 23.9% | 20.3% | 12.3% | 8.2% | 0.2% | 0% |

**Table S3: Number of SE genes detected using different kernels.** We paired SPARK-X with each of the 11 kernels constructed based on either the original coordinates (Projection), each of the five Gaussian coordinates (Gaussian1-5) or each of the five Cosine transformed coordinates (Cosine1-5), in each of the five datasets analyzed (columns). Rows 1-11 display the numbers of SE genes detected by each kernel. Inside the parenthesis show the number of SE genes detected uniquely by each kernel. The 12^th^ row displays the number of SE genes detected in the original SPARK-X that is paired with all 11 kernels.

| **Data Set** | **Mouse Cerebellum (SlideseqV1)** | **Mouse Cerebellum (SlideseqV2)** | **Mouse**  **Olfactory Bulb**  **(HDST)** | **Human Heart (Visium)** | **Human Ovarian Cancer**  **(Visium)** |
| --- | --- | --- | --- | --- | --- |
| **Projection** | 574 (105) | 256 (113) | 29 (8) | 1316 (605) | 154 (18) |
| **Gaussian1** | 773 (6) | 232 (7) | 9 (0) | 251 (5) | 460 (0) |
| **Gaussian2** | 1708 (1) | 466 (0) | 65 (0) | 721 (1) | 227 (0) |
| **Gaussian3** | 2148 (0) | 472 (0) | 100 (0) | 739 (0) | 184 (0) |
| **Gaussian4** | 2163 (0) | 420 (0) | 98 (0) | 715 (0) | 178 (0) |
| **Gaussian5** | 2131 (0) | 329 (0) | 86 (0) | 689 (0) | 180 (0) |
| **Cosine1** | 0 (0) | 0 (0) | 0 (0) | 0 (0) | 0 (0) |
| **Cosine2** | 9 (0) | 1 (0) | 5 (0) | 1 (0) | 20 (0) |
| **Cosine3** | 135 (17) | 25 (3) | 7 (0) | 74 (1) | 268 (3) |
| **Cosine4** | 47 (1) | 69 (6) | 66 (3) | 532 (33) | 553 (35) |
| **Cosine5** | 107 (4) | 224 (4) | 30 (3) | 31 (1) | 512 (6) |
| **SPARK-X** | 2336 | 688 | 125 | 1536 | 651 |
