# Supplementary material for: SPARK-X: non-parametric modeling enables scalable and robust detection of spatial expression patterns for large spatial transcriptomic studies
Source: Genome Biol. 2021 Jun 21;22:184. doi: 10.1186/s13059-021-02404-0 (PMC8218388; doi:10.1186/s13059-021-02404-0)
Supplement: Supplementary file 6 — Additional file 6. Review history. [file 13059_2021_2404_MOESM6_ESM.docx]

Review History

**First round of review**

**Reviewer 1**

**Were you able to assess all statistics in the manuscript, including the appropriateness of statistical tests used?**

Yes: The paper provides rigorous assessment on the type-I errors and powers of the proposed tests, as well as other alternative methods in both the simulation examples and real data examples.

**Were you able to directly test the methods?**

No

**Comments to author:**

The paper introduced a novel method to search for genes with spatial expression patterns based on spatial transcriptomic data. The authors used the idea of distance-correlation and proposed a non-parametric test to assess the concordance between sample (cell/spot) covariance based on the expression levels of a given gene across all cells/spots and that based on the spatial locations of these cells/spots. The idea is intuitive and effective. The advantage of the proposed method over existing tools on sparse ST data is convincing: better controlled type-I error, improved power, and dramatically improved computational efficiency. Also, the method can be applied to 3D or even higher dimensional ST data sets (e.g. with a time dimension), which is a good plus. This work is very timely, and would have broad applications in spatial transcriptomic based research.

I only have a few minor questions/comments:

1. SPARK-X uses one "covariance" measurement for expression data, while 10 different "covariance" measurements for spatial location based on 10 different transformations of the x/y-coordinates.

a. The motivation and effects of the 10 different transformations is not provided. It will be helpful if the authors can further elaborate these aspects in the paper.

b. Any scaling/centering is needed for the x/y-coordinate before performing the transformation?

c. The parameters in the transformation "are predetermined based on quantiles of the x and y coordinates in the data". Please provide more details/justifications for these choices.

2. Sometimes, there is a need to look for genes with spatial differential expression patterns in a particular type of cells. In that situation, the location of the cells of a certain type could form some irregular or even disconnected shapes/segments, instead of a uniform spread over a large region. How will SPARK-X perform under that situation? Will a special "transform" of spatial coordinate needed to ensure the effectiveness of SPARK-X?

3. When sparsity is moderate (average 62.1% zero), SPARK appeared to have well controlled type-I error, and comparable or even better power compared to SPARK-X (Figure S2/S3). On the other hand, the authors pointed out that SPARK does not work when sparsity in the data is high (99.5%).

a. Is the problem of SPARK encountered when the data is sparse but small?

b. Will the break-down of SPARK happen when the sparsity is not that extremely, say at 90% or 80%? How will SPARK-X compared to SPARK under those settings?

**Reviewer 2**

**Were you able to assess all statistics in the manuscript, including the appropriateness of statistical tests used?**

Yes: The statistics in the manuscript are appropriate.

**Were you able to directly test the methods?**

Yes

**Comments to author:**

This paper proposes a new method, SPARK-X, for scalable detection of genes with spatial-expression pattern from large scale spatial transcriptomics data. The method is quite different from the earlier version of their software, SPARK. This new approach can potentially be very innovative (if the confusions I list below are cleared), and it is presented in the paper with compelling examples and impressive benchmarking results. However, there are many aspects of the method that, as written, I find unintuitive. Since this is a methods paper, the reader would benefit from more careful presentation of the intuitions and rationale of the model, and not just the benchmarking results. I feel that I can't really believe the benchmarking results, or judge the merits of the method, until the concerns below are addressed.

Concerns/Suggestions:

On Method:

1. Page 12 lines 46 - 57: As written, this method is very unintuitive, especially for the biological audience. First, what you call a "covariance matrix" is not really what we usually think of as the covariance. Your y is 1xn. Normally, if y is nxk and columns sum to 0, then y'y is the covariance. How is (yy')/(y'y), as defined, a covariance matrix?

2. Still on the model in lines 46-57: It seems that you mainly want to get a nxn matrix, where the (i,j)-th entry is some sort of "similarity" between the i-th and j-th pixel, correct? As written, that similarity is computed as y_i times y_j, which rewards situations when y_i and y_j are both very negative or very positive. But is it true that you want the "reward" to scale in this way? For example, if y_i=1, y_j=1/(y'y), this similarity as defined would be 1, which is smaller than if y_i=1 and y_j=10. That doesn't make sense to me.

3. Still on the model in lines 46-57: You also compute a similar "covariance" for the positions: \Sigma=SS'/((S'S). Again, how is this a covariance? It seems you want an nxn matrix that captures the "closeness" between the pixels, is that correct? In the \Sigma matrix, pixel pairs that have the same distance to each other are not equally rewarded. It seems that you are rewarding proximity between pixels more when the pixels are at the edges of the image, is that correct? Why would this be desirable?

On overall focus:

I find that the problem of finding genes with "spatially differentiated" transcription patterns to be the first step, but not that exciting, because most genes that are differentially expressed across cell types would be spatially differentiated, because cell types are never randomly mixed in tissues. Thus, the default version of your method would simply be finding cell type markers, as you are well aware, since you use cell type markers to validate the method. It is fine that you start with this perspective, but, a more interesting question would be "what genes are spatially differentiated, after accounting for cell type?" your proposed method does allow us to probe at this question, and you touched upon it in your second data example (mouse cerebellum, Slide-seqV2). I feel this paper would be stronger if you stress this component more and add perhaps one more example of what this type of conditional analysis brings us.

Minor comment: In line 43: "detected by SPARK-X", is this a typo?

**Reviewer 3**

**Were you able to assess all statistics in the manuscript, including the appropriateness of statistical tests used?**

Yes: Appropriate statistical methods

**Were you able to directly test the methods?**

No

**Comments to author:**

Yhu J. et al provide a novel method for the analysis of spatial transcriptomic (ST) data called SPARK-X. The tests made to evaluate the tool performance are appropriate and overall it provides a robust method for the identification of genes associated with spatial patterns. Although the improvements on the tool are significant, these are mostly technical and there is no real biological knowledge gained from their analyses, which are limited to brain tissue. We would like to recommend to the authors to improve on their biological interpretation of SPARK-X results or consider sending their work to a more adequate journal.

Major comments:

The authors should reconsider the name of the tool: https://www.dictionary.com/browse/expediate.

The phrase "dozens to thousands of GB physical memory" is not appropriate. Also some people use physical memory as to mean both RAM and hard drives (like here: Difference Between Physical and Virtual Memory | Compare the Difference Between Similar Terms ). So maybe "physical RAM memory" on this first instance to clarify this.

Although the authors provide a proper evaluation of their methods on the main current ST platforms, they limit themselves to using only brain samples. It would be really beneficial for their work to include other tissues that are also publicly available.

The authors need to include an example using 10X Genomics Visium data, which is becoming more widely used due to its commercialisation.

Minor comments:

The line numbering restarts on each page, which makes the reviewing process a bit difficult.

The authors' statement "trendsceek[4], SpatialDE[5], and SPARK[6]. Unfortunately, even the latter two computationally efficient methods are not readily applicable for analyzing large-scale sparse spatial transcriptomic data", relies on trendsceek being known as computationally inefficient - is this backed up by any evidence?

Can the authors comment on what Bayesian alternatives could be used as an alternative to p-values?

Spatial expression patterns include hotspot, streak, and gradient. It might be worth writing a comment (in the discussion perhaps?) about how much this covers geometrical patterns found in real images and to what extent other patterns (like circular gradient) would be picked up.

**Authors Response**

**Point-by-point responses to the reviewers’ comments:**

Reviewer: 1#

General Comments:

The paper introduced a novel method to search for genes with spatial expression patterns based on spatial transcriptomic data. The authors used the idea of distance-correlation and proposed a non-parametric test to assess the concordance between sample (cell/spot) covariance based on the expression levels of a given gene across all cells/spots and that based on the spatial locations of these cells/spots. The idea is intuitive and effective. The advantage of the proposed method over existing tools on sparse ST data is convincing: better controlled type-I error, improved power, and dramatically improved computational efficiency. Also, the method can be applied to 3D or even higher dimensional ST data sets (e.g. with a time dimension), which is a good plus. This work is very timely, and would have broad applications in spatial transcriptomic based research.

*Response: Thank you for the positive comments and constructive suggestions, which have substantially improved the quality of the manuscript. Our detailed responses to your specific comments are listed below.*

Specific Comments:

1.	SPARK-X uses one "covariance" measurement for expression data, while 10 different "covariance" measurements for spatial location based on 10 different transformations of the x/y-coordinates.

a.	The motivation and effects of the 10 different transformations is not provided. It will be helpful if the authors can further elaborate these aspects in the paper.

*Response: Thank you for the comment. Following your suggestion, we have included a detailed motivation for the use of 10 different transformations. Specifically, the different transformations are used to capture different spatial expression patterns one might encounter in real data applications. In particular, the kernel constructed based on the untransformed coordinates is useful to detect linear expression pattern across the coordinates. The kernels constructed based on the cosine transformed coordinates are useful to detect periodic expression patterns on the tissue. While the kernels constructed based on the Gaussian transformed coordinates are useful to detect focal expression patterns on the tissue. Therefore, combining the original and transformed distance covariance matrices would allow us to detect a wide variety of spatial expression patterns encountered in real data. We illustrate the kernels constructed based on different transformations in Fig.S17 in the form of heatmaps, which capture the spatial expression pattern each given kernel is geared towards detecting. We have added the above motivation in the Materials and Methods section (lines 489-494 on page 14; lines 515-521 on pages 14-15).*

*To further illustrate the effects and benefits of using different transformations, we performed additional analysis in all five real data applications. Specifically, we paired SPARK-X with one kernel at a time to analyze each data and examined the number of SE genes detected by each kernel. The results are listed below for convenience (Rev1.Table1) and are included in the new Supplementary Table S2 in the updated manuscript. In the analysis, we found that the number of detected SE genes varies across kernels and that different kernel has different power in different data sets. However, by combing the association evidence across all kernels, the original SPARK-X achieves higher power than each individual kernel. Therefore, the use of 10 different transformations in SPARK-X ensures robust identification of SE genes with various spatial patterns across data sets. We have added these new results in the Discussion section (lines 381-386 on page 11).*

*Rev1. Table1: Number of SE genes detected in the five real datasets using different kernels. We paired SPARK-X with each of the 11 distance covariance matrices constructed based on either the original coordinates (Projection), each of the five Gaussian coordinates (Gaussian1-5), or each of the five Cosine transformed coordinates (Cosine1-5), and analyzed the five datasets (columns). Rows 1-11 display the numbers of SE genes detected by each kernel. Inside the parenthesis show the number of SE genes detected uniquely by each kernel. The 12th row displays the number of SE genes detected in the original SPARK-X that is paired with all 11 kernels. The five datasets include: SlideseqV1 (SV1), SlideseqV2 (SV2), High-Definition Spatial Transcriptomics (HDST); 10X Visium Human Heart (HH_10X), and 10X Visium Ovarian Cancer (OC_10X).*

Any scaling/centering is needed for the x/y-coordinate before performing the transformation?

*Response: Thank you for the comment. Scaling the x/y-coordinates before the transformation does not influence the results because (1) the scale of the coordinates is appropriately accounted for in the covariance test statistics as it includes a normalizing factor (S^T S)^(-1), and this normalizing factor effectively makes the test statistics invariant to scale transformation; and (2) the kernel parameters used in the Gaussian and cosine transformations are obtained based on the quantiles of the data, which makes the resulting kernels also scale invariant. Centering, on the other hand, does affect the resulting kernels and thus the transformation results. Indeed, centering will likely influence statistical power, even though it would not affect type I error control as we can still obtain the exact distribution of the covariance test statistics under the null regardless how the kernel matrices are constructed. While we previously did not perform centering, we believe that centering would likely help improve the statistical power as it would allow us to focus the analysis onto the center of the tissue section. Therefore, following your comments, we decided to re-analyze all of our data, both simulations and real data applications, using the centered (and scaled) version of the coordinates. Because of centering, we detected slightly more SE genes with SPARK-X in the updated manuscript, though all the previous conclusions remain the same in the real data. In the simulations, the power of SPARK-X also increases, especially for the hotspot pattern. It now has a comparable power as SPARK in detecting the hotspot pattern across all different settings (Fig.S2), while remains more powerful than SPARK in detecting the other patterns. We have updated our software to set centering and scaling as the default option. We have updated the method details in the Materials and Methods section (lines 409-411 on page 13) and the results in the Results section accordingly to reflect these changes. All conclusions remain the same.*

The parameters in the transformation "are predetermined based on quantiles of the x and y coordinates in the data". Please provide more details/justifications for these choices.

*Response: Thank you for the comment. We primarily followed the main ideas of SpatialDE[1] and SPARK[2] in using the empirical quantiles of the data to construct different covariance matrices. Compared to the alternative approach of fixing the transformation parameters to some pre-determined values, using the quantiles of the data for transformation has the benefits of being invariant to any scale transformation of the original data and allows us to construct the distance covariance matrices in a data dependent fashion. We have added these motivations and justifications both before (lines 489-494 on page 14) and after (lines 507-511 on page 14) the previous details on how the transformation parameters were determined based on data in the updated Materials and Methods section.*

*In addition, we also performed additional analysis to examine whether the results are robust with respect to different choices of the transformation parameters. Specifically, in addition to using the five quantiles used in the previous version of the manuscript (20%,40%,60%,80%, and 100%), we also examined the use of ten quantiles that range from 10% to 100% in the simulations in the two sparsity simulation settings (zero proportions set to be either 80% or 90%). In the new analysis, we effectively paired SPARK-X with 21 different kernels (one original; ten for Gaussian transformation and then for Cosine transformation) as compared to the previous SPARK-X that was paired with 11 kernels. In the analysis, we found that the results obtained based on the ten quantiles were almost identical as the results obtained using five quantiles. We provide these new results in the following figure for your reference (Rev. Figure 1).*

*Rev1. Figure 1. Power comparison between two versions of SPARK-X, one with five data quantiles for the transformation of the coordinates and one with ten data quantiles for the transformation of the coordinates. Power (y-axis) are shown in the two simulation settings with 80% zero proportions (lower) or 90% zero proportions (upper), across three different samples sizes (SS=500, 1,000, 2,000) and two expression patterns (streak and hotspot). The other parameters used in the simulation settings are dispersion = 0.2, foldchange=2, fraction of marked cells = 0.2. The overlap of detected SE genes between the two versions of SPARK-X is also high. For example, in the simulations with 80% sparsity level and the largest sample size (SS=2000), the median Jaccard Index is 0.96 for the streak pattern and 0.98 for the hotspot pattern.*

2.	Sometimes, there is a need to look for genes with spatial differential expression patterns in a particular type of cells. In that situation, the location of the cells of a certain type could form some irregular or even disconnected shapes/segments, instead of a uniform spread over a large region. How will SPARK-X perform under that situation? Will a special "transform" of spatial coordinate needed to ensure the effectiveness of SPARK-X?

*Response: Good point. Following your comments, we performed new analyses on the Slide-seqV2 data on two cell types that display highly irregular and somewhat disconnected shapes on the tissue. Specifically, we rely on the cell type inference results from RCTD to extract the locations dominated by the Purkinje cells and the locations dominated by the granular cells. The Purkinje cells are primarily located in the thin Purkinje cell layer while the granule cells are located in the thick granular layer; both layers are of highly irregular shapes. After removing the genes with no expression on any of the selected cells, we analyzed 3,006 genes on 652 Purkinje cells and 3,288 genes on 5,891 granule cells, in two separate analyses. In the analyses, we found that the permuted p-values for both cell types are generally well behaved. SPARK-X detected 3 SE genes for Purkinje cells and 249 SE genes for granule cells. All these genes show clear spatial expression pattern within each cell type. We list a few of them for each cell type in Rev. Figure 2. For example, the two cerebellum compartmentalization genes, Aldoc for Purkinje cells and Ptprt for granule cells, are both detected as showing SE pattern within each cell type. In addition, Kctd12 shows similar pattern as the Aldoc within the Purkinje cells, while Mapped2 shows enriched expression towards the posterior lobes. Most SE genes detected in the granule cells (e.g. Rasgrf1 and Ttr as shown below) also show high expression in certain lobes and low expression in the others. Overall, SPARK-X appears to be effective in detecting cell type specific expression patterns with irregular shapes without the need of performing additional special transformations on the coordinates. We have described the results in the updated Results section (lines 243-246 on page 7), with the results showing in the new Fig.S9, which is also included below for convenience.*

*Rev1. Figure 2 Cell type specific SE analysis with SPARK-X. Cell type specific analysis is performed on either Purkinje cells (A-B) or granule cells (C-D) in the Slide-seqV2 cerebellum data. (A, C). Quantile–quantile plot of the observed −log10(P) against the expected −log10(P) under the permuted null in the corresponding cell type. P values were combined across ten permutation replicates. Spatial expression pattern of representative SE genes detected by SPARK-X are shown for the Purkinje cell specific analysis (B) and granule cell specific analysis (D). In both B and D, the background antique white color represents cells that belong to the analyzed cell type.*

3.	When sparsity is moderate (average 62.1% zero), SPARK appeared to have well controlled type-I error, and comparable or even better power compared to SPARK-X (Figure S2/S3). On the other hand, the authors pointed out that SPARK does not work when sparsity in the data is high (99.5%).

a.	Is the problem of SPARK encountered when the data is sparse but small?

*Response: Thank you for the comment. The algorithm stability and convergence issue encountered by SPARK is due to the sparsity of the data and does not appear to be related to the sample size. This algorithmic issue is not restricted to SPARK and generally applies to fitting algorithms for the generalized linear models such as the negative binomial distribution. For example, we applied the negative binomial model implemented in the glm.nb function in the MASS R package to analyze the data and encountered similar algorithm stability and convergence issue. Following your comment, we have performed additional simulations with different sparsity levels to more carefully characterize this algorithm issue with count models. With the new analyses, we observed that the algorithmic stability issue starts to show up for SPARK when the sparsity level in the data is above 97%. Similar stability issue starts to show up for the negative binomial model when sparsity is above 90%. We have added these results to the new Supplementary Table 3 with results details described in the Results section (lines 107-112 on page 4). We also provide these results below for your convenience (Rev1. Table 3).*

*Rev1. Table 3: Percentage of failures in SPARK and the negative binomial model in simulations with different data sparsity levels. The negative binomial model is implemented in glm.nb in the MASS R package. We set maxit to be 500 in glm.nb. The failure percentage was calculated based on the number of failures in 1,000 simulation replicates. In the simulation, we varied the mean parameter of the negative binomial distribution (0.005, 0.01,0.02,0.03,0.04,0.05,0.1,0.2) to generate data with different sparsity levels. The sample size is set to be 3000 and the dispersion parameter is set to be 0.2.*

b.	Will the break-down of SPARK happen when the sparsity is not that extremely, say at 90% or 80%? How will SPARK-X compare to SPARK under those settings?

*Response: Thank you for the comment. As shown in the above table, the algorithm for fitting SPARK is stable when the sparsity level is 90 % or 80%. Therefore, we performed new simulations with non-extreme sparsity levels to compare SPARK with SPARK-X following your comment. The results from the new simulations are quite consistent with our previous simulations results (Fig.S2) and suggest that SPARK-X is more powerful than SPARK in detecting the streak pattern and comparably powerful in detecting the hotspot pattern (Fig.S2). We also recognize that the sparsity level of some of the 10X genomics data are non-extreme. Therefore, we obtained two 10x genomics data including a human heart data (with a sparsity level of about 90%) and a human ovarian cancer data (which was collected through an enriched protocol and thus came with a very low sparsity level of about 74%). In both datasets, SPARK-X detects more SE genes than SPARK (Figs. S12 and S13). Because SPARK is based on a generalized linear mixed model, its computation burden increases cubically as sample size increases (as compared to linear complexity of SPARK-X). Therefore, we can only perform comparison between SPARK and SPARK-X in simulations and real datasets with relatively small sample sizes. The new real data results are shown in Figs. S12 and S13, with details described in the updated Discussion section (lines 342-350 on page 10). We did not include the new simulation results to the updated manuscript but listed them below for your reference (Rev1. Figure 3).*

*Rev1. Figure 3. Power comparison of SPARK-X and SPARK in the 80% sparsity setting and 90% sparsity setting. Power (y-axis) are shown in the two simulation settings with 80% zero proportions (lower) or 90% zero proportions (upper), across three different samples sizes (SS=500, 1,000, 2,000) and two expression patterns (streak and hotspot). The other parameters in the simulations are set to be dispersion = 0.2, foldchange=2, and fraction of marked cells = 0.2.*

------------------

Reviewer: 2#

General Comments:

This paper proposes a new method, SPARK-X, for scalable detection of genes with spatial-expression pattern from large scale spatial transcriptomics data. The method is quite different from the earlier version of their software, SPARK. This new approach can potentially be very innovative (if the confusions I list below are cleared), and it is presented in the paper with compelling examples and impressive benchmarking results. However, there are many aspects of the method that, as written, I find unintuitive. Since this is a methods paper, the reader would benefit from more careful presentation of the intuitions and rationale of the model, and not just the benchmarking results. I feel that I can't really believe the benchmarking results, or judge the merits of the method, until the concerns below are addressed.

*Response: Thank you for the comments and constructive suggestions, which have substantially improved the quality of the manuscript. Our detailed responses to your specific comments are listed below.*

Specific Comments:

1.	Page 12 lines 46 - 57: As written, this method is very unintuitive, especially for the biological audience. First, what you call a "covariance matrix" is not really what we usually think of as the covariance. Your y is 1xn. Normally, if y is nxk and columns sum to 0, then y'y is the covariance. How is (yy')/(y'y), as defined, a covariance matrix?

*Response: Thank you for the comment. We refer to the matrix (yy')/(y'y) as the covariance matrix because it is generated from the projection covariance function and possesses the two key covariance matrix properties including being (a) symmetric and (b) positive semi-definite. In statistics and machine learning literature, a covariance matrix is often used interchangeably with a kernel matrix, and a covariance function is used interchangeably with a kernel function. The projection covariance function does not require the input y to have a dimension higher than one and does not require y to be centered. The projection covariance function has been widely used in many applications in statistics, machine learning and genetics[3-5].*

*We did not explicitly center y in the previous version of the manuscript because the covariance test applies to any covariance kernel function and that the projection covariance function does not require the input of y to be centered. While we did not explicitly center y during the construction of the projection covariance kernel, we note that the covariance test implicitly centered y when computing the test statistics. In particular, the covariance matrix is multiplied by a hat/projection matrix H on both sides to yield the final test statistics (lines 432-438 on pages 12-13). Multiplying H on y effectively centers y to have a mean of zero. Therefore, we effectively centered y in constructing the covariance test in the previous version of the manuscript. To avoid future confusions on centering vs non-centering, we have updated all analyses with the centered version of y and S and updated the text accordingly.*

*Your specific comment appears to describe a sample covariance matrix, which is certainly obtained with multiple samples (i.e. k>1) and requires explicitly centering of each element of y (based on n different means). The covariance test does not use a sample covariance matrix. We have added clarifications on this importance issue of why this is called a covariance matrix in the updated method overview section (lines 427-432 on page 12).*

2.	Still on the model in lines 46-57: It seems that you mainly want to get a nxn matrix, where the (i,j)-th entry is some sort of "similarity" between the i-th and j-th pixel, correct? As written, that similarity is computed as y_i times y_j, which rewards situations when y_i and y_j are both very negative or very positive. But is it true that you want the "reward" to scale in this way? For example, if y_i=1, y_j=1/(y'y), this similarity as defined would be 1, which is smaller than if y_i=1 and y_j=10. That doesn't make sense to me.

*Response: Thank you for the comment. We are a bit puzzled by your comment here, given that what you mentioned as “doesn’t make sense” is both the standard property of the projection covariance function and the standard property of the sample covariance you suggested in comment #1. Specifically, the sample covariance, by definition, is computed based on the product of the centered versions of y_i and y_j. The sample covariance aims to capture the coordinated deviations from the mean and thus rewards situations where y_i and y_j are both very negative or very positive (i.e. with large deviations from the mean of zero in the same direction). In your particular example, the contribution to the sample covariance would be higher for y_i=1 and y_j=10 as compared to y_i=1 and y_j=1, as the former provides stronger evidence on the coordinated deviations from the mean for these two locations. Similarly, the projection kernel function we use in the covariance test also measures the coordinated deviation from the mean[3, 4].*

*The coordinated deviation from the mean is commonly used as a similarity measure in many kernel functions other than the projection kernel. For example, the sigmoid kernel[6], the polynomial kernel[7], and the wavelet kernel[8] all perform different linear or non-linear transformation on the product of two input values. Certainly, the product of two input values is only one similarity measurement. Other similarity measurements include the Euclidean distance between two input values as used in the Gaussian kernel, the minimum of two input values as used in the histogram intersection kernel[9, 10], and the minimum of power functions of the two input values as used in the generalized histogram intersection kernel[11], among many others[12-14]. Different kernels describe different notions of similarity and some of these similarities may not be easily interpreted in an intuitive way [13-15].*

*The similarity measurement obtained through the projection covariance function can be particularly useful when the gene expression shows a gradient pattern along one axis. In addition, our simulations and real data applications also show that the projection covariance function is reasonably robust to detect a range of other spatial expression patterns. We certainly acknowledge that other kernels that use different similarity metrics can be potentially beneficial in capturing other expression patterns. For example, by using a Euclidean distance, the Gaussian kernel used in SPARK can be particularly useful in capturing focal expression patterns. Importantly, the covariance test is general and can be paired with any kernel function that measure different expression similarity between pairs of locations to take advantage of their benefits.*

*Based on your comments, we suspect that our previous version of the manuscript leads to this unfortunate miscommunication because we failed to explain well the term “similarity”. “Similarity” has a broad definition in the statistics and machine learning literature[15-18] but can give out a false narrow impression that it has to be measured using Euclidean distance only. Following your comment, we have added a short clarification on what we mean by “similarity” for this particular covariance function in the Method overview section (lines 439-440 on page 13). In addition, we have added a new paragraph of discussion on this important issue of “similarity” and use of different kernels in the Discussion section (lines 366-388 on page 11).*

3.	Still on the model in lines 46-57: You also compute a similar "covariance" for the positions: \Sigma=SS'/((S'S). Again, how is this a covariance? It seems you want an nxn matrix that captures the "closeness" between the pixels, is that correct? In the \Sigma matrix, pixel pairs that have the same distance to each other are not equally rewarded. It seems that you are rewarding proximity between pixels more when the pixels are at the edges of the image, is that correct? Why would this be desirable?

*Response: Thank you for the comment. As we explained in the response to your first comment, \Sigma=SS'/((S'S) is a covariance matrix because it is generated from the projection covariance function and has the standard covariance matrix properties including being symmetric and positive semi-definite. Also, as we explained in the response to your second comment, the projection kernel (and the sample covariance) measures similarity in terms of coordinated deviations from the mean/center.*

*With the projection covariance function, the pixel pairs that have the same Euclidean distance to each other are not equally rewarded. For example, as the reviewer correctly pointed out, the pixels at the edges of the image will tend to have large values in the distance covariance matrix. Emphasizing on the edges of the image is optimal for detecting spatial expression patterns on the edges of the image or patterns that display a gradient from the center to the edge. For example, the power of SPARK-X is especially high for detecting the gradient pattern in the simulations as illustrated there. On the other hand, emphasizing the edges of the image is likely not optimal on detecting spatial expression patterns in the center of the image or focal expression patterns. However, as the simulations and the real data results suggest, the projection covariance function is quite robust in detecting a range of possible spatial expression patterns. For example, in simulations, the power of SPARK-X in detecting the hotspot pattern remains high as compared to the other methods. SPARK-X also detected many genes that show focal or irregular patterns in the real data, such as Kcnc3 in Fig. 2F and Aldoc in the Fig. 3D.*

*Certainly, no kernel is optimal in detecting all spatial expression patterns. Because the covariance test is general, it can be paired with any kernel function that is optimal in detecting certain spatial expression patterns. Indeed, we have created various transformation of the coordinates in order to construct different covariance matrices to capture a wide variety of spatial expression patterns, including the periodical patterns (Fig. S17). Importantly, the projection kernel allows us to achieve orders of magnitude of computational gains and orders of magnitude memory savings as compared to other kernels such as the Gaussian kernel. Therefore, we hope the reviewer would agree that the benefits of using the projection kernel in the covariance test clearly outweighs its drawbacks in terms of a potential power loss in detecting certain spatial expression patterns.*

*We have updated the method overview section to clarify these important points in line of the paragraphs above (lines 427-432 on page 12; 489-494 on page 14; lines 515-521 on pages 14-15). We have also added a paragraph of discussion on the use of different kernels in the Discussion section (lines 366-388 on page 11).*

*Finally, we recognize that your first three comments are all centered on the intuitions behind the covariance test. We agree with the reviewer that the general covariance test originally described on the theoretical statistical literature is not an intuitive method and that the kernel functions commonly used in machine learning literature may not be easy to describe in an intuitive way towards a general audience. We have tried previously to describe the covariance test in the setting of SE analysis towards a general audience without going into the theoretical details of the method. Based on your comments, we realized our previous description was insufficient and potentially caused several miscommunications. Following your comments, we have modified the method overview section to clarify these important miscommunications, with the hope that the method description is now more accessible to the general audience. For example, we have included additional intuitions on how the covariance test statistics can be used to test the independence between y and S (lines 439-447 and lines 453-456 on page 13). Please refer to the updated Method overview section for details (pages 12-15).*

4.	I find that the problem of finding genes with "spatially differentiated" transcription patterns to be the first step, but not that exciting, because most genes that are differentially expressed across cell types would be spatially differentiated, because cell types are never randomly mixed in tissues. Thus, the default version of your method would simply be finding cell type markers, as you are well aware, since you use cell type markers to validate the method. It is fine that you start with this perspective, but a more interesting question would be "what genes are spatially differentiated, after accounting for cell type?" your proposed method does allow us to probe at this question, and you touched upon it in your second data example (mouse cerebellum, Slide-seqV2). I feel this paper would be stronger if you stress this component more and add perhaps one more example of what this type of conditional analysis brings us.

*Response: Thank you for the comment. Following your suggestion, we have attempted to perform such conditional analysis on the other two real data examples (Slide-seq data and HDST data). The conditional analysis is technically difficult, however, as it requires cell type annotations or cell type composition estimates on each spatial location of the assayed tissue. These cell type composition information are not readily available and are often challenging to obtain using existing bioinformatics tools. Specifically, when we applied RCTD to the first data (Slide-seq data) to perform cell type deconvolution, RCTD only succussed in a small proportion of measured locations (4,548 out of 25,551 beads), many of which are disconnected from each other. The failure of RCTD in the first data is presumably because of the high sparsity of the data (99.4% zeros). Because we cannot obtain cell type composition estimates for majority of locations using RCTD, we are unable to perform conditional analysis in the first data. In addition, RCTD failed to extract cell type composition estimates in the third data (HDST data) because RCTD is not computationally scalable to that data.*

*Fortunately, the HDST data is of approximately single cell resolution. Therefore, we directly obtained the cell type labels from the original paper and performed SE analysis by including the cell labels as covariates. In the analyses, SPARK-X identified 66 and 36 SE genes (out of a total of 17,121 genes) with and without controlling for cell types, respectively (overlap = 35). Careful examination on the detected SE genes suggests that SE genes that remained significant after controlling for cell types often display spatial expression pattern across multiple cell types or within the same cell type (Fig. S11E and S11F). For example, Camk1d is enriched in the ventral part of multiple olfactory bulb layers including the external plexiform layer and the glomerular layer. It is specifically enriched in a subset of inhibitory neurons that reside outside the granule cell layer. We have included these new conditional analysis results in the new Fig.S11. The analysis procedure is provided in the Materials and Methods section (lines 654-661 on pages 17-18) and the corresponding results are described in the updated Results section (lines 287-312 on page 8).*

*Besides the new conditional analysis, we also followed the essence of your comment and performed new cell type specific analysis in both the SlideseqV2 data and the HDST data. Specifically, we applied SPARK-X to analyze spatial locations that are dominated by Purkinje cells or granule cells in the SlideseqV2 data and analyze the inhibitory neurons in the HDST data. These cell type specific analyses allow us to directly detect many SE genes that display spatial expression pattern within a given cell type. The analysis procedure is provided in the Materials and Methods section (lines 631-637 on page 17, lines 663-668 on page 18). The new results are shown in Fig. S9 and S11 and are described in the updated Results section (lines 243-246 on page 7; lines 300-312 on pages 8-9). We have also added sentences to the abstract (lines 20-21 on page 1) and the introduction (line 77 on page 3) to stress the results from the conditional and cell type specific analyses. We hope that the new set of conditional analysis and these new sets of cell type specific analyses make this manuscript stronger.*

5.	Minor comment: In line 43: "detected by SPARK-X", is this a typo?

*Response: Thank you for catching the typo. We have corrected it to “detected by SPARK-G” (line 205 on page 6).*

Reviewer: 3#

General Comments:

Zhu J. et al provide a novel method for the analysis of spatial transcriptomic (ST) data called SPARK-X. The tests made to evaluate the tool performance are appropriate and overall it provides a robust method for the identification of genes associated with spatial patterns. Although the improvements on the tool are significant, these are mostly technical and there is no real biological knowledge gained from their analyses, which are limited to brain tissue. We would like to recommend to the authors to improve on their biological interpretation of SPARK-X results or consider sending their work to a more adequate journal.

Response: Thank you for the positive comments and constructive suggestions. Our detailed responses to your specific comments are listed below. Here, we would like to take the opportunity to address your general comment on “no real biological knowledge gained” and “improve on their biological interpretation of SPARK-X results” since none of the following specific comments appears to focus on this important issue.

*First, we note that our manuscript is submitted to the journal as a Method paper, not as a Research article. A method paper is in a slightly different format as a research article (e.g. only 100 words in the abstract as compared to 250) and is judged differently based on a different set of criteria as compared to a research article. As stated on the journal website, the key criterion for judging the suitability of a genomic method paper is to “describe novel methods that are shown to be a clear advance over existing state-of-the-art methods in a side-by-side demonstration”. Indeed, the editor explicitly stated in the decision letter to us that “we wouldn’t require novel biological findings in order to publish the paper”.*

*Second, we understand that “real biological knowledge” and “biological interpretation” are subjective terms and different researchers in different fields may have completely different view on what constitute “real biological knowledge” and “biological interpretation”. As a method paper, we believe that we have identified, described and interpreted many novel biological findings, at least way beyond what were typically expected and reported in a method paper published on a similar journal. Perhaps it helps to put this statement into context. Let’s look at the two of the most highly cited genomic method papers for the previous differential expression (DE) analysis: DESeq[19] (Genome Biology, 2010) and DEseq2[20] (Genome Biology, 2014). Both these DE method papers carried out method comparisons using simulations and reported only the number of DE genes detected in the real datasets without examining the biological interpretation of any identified DE genes. Similarly, the majority of DE method papers published in Genome Biology in the past five years only performed power comparisons based on the number of detected DE genes with occasional additional gene set enrichment analysis [21-25]. Given this context, we believe our manuscript produces substantially more “new biological knowledge” and describes more “biological interpretation” than a typical method paper published on a similar journal. In particular, we analyzed five real datasets (which is a lot more than that in a typical method paper), identified way more SE genes than existing SE approaches on all these data, and recorded orders of magnitude computational gains as compared to the existing approaches. In addition, we described in detail the spatial expression patterns of many SE genes, the implied transcriptomic mechanisms underlying the functional organization of the examined tissues, and, through functional enrichment analysis, revealed new biological pathways that underly the tissue functional and transcriptomic architectures.*

*Third, we note that our study discovered many novel SE genes that cannot be identified by any existing methods on the same data. A careful examination of these discovered SE genes further revealed many new biology that cannot be discovered by existing methods. For example, in the SlideseqV1 data, only SPARK-X identified the SE gene Cbln1, which is functionally important for synaptic organization in the cerebellum. In the SlideseqV2 data, only SPARK-X identified the SE genes Ptprt and Aldoc, both of which show spatial expression pattern only in a subset of the granule cells or the Purkinje cells, thus potentially representing the transcriptional mechanism underlying the cerebellum compartmentalization. In the HDST data, only SPARK-X identified the SE genes Gphn and Dlg2, which display spatial expression pattern that imply their critical roles in the organization of lateral inhibition in the olfactory bulb.*

*Fourth, our method enables biologists to make future biological discoveries on large-scale spatial transcriptomics datasets that are becoming increasingly popular. Many of these discoveries are simply not possible without our method. Specifically, our method brings in huge improvement in statistical power that is critical for detecting new SE genes, which are key for understating the transcriptomic landscape of complex tissues. In addition, and equally importantly, our method brings several orders of computational gains as compared to existing approaches, making it the only SE method applicable to large-scale spatial transcriptomics studies such as the HDST examined here. Large-scale spatial transcriptomics studies are key for characterizing the functional and architectural organization of tissues and are key for revealing new biological mechanisms underlying various development processes and disease etiology at the tissue level.*

*Finally, we followed the essence of your general comment and performed new analyses to gain additional biological knowledge with additional biological interpretation. Specifically, we performed conditional analysis to identify SE genes that display spatial expression patterns that are independent of the cell types (lines 287-313 on pages 8-9; Fig.S11). We performed cell specific analysis to identify SE genes that display spatial expression pattern within the same cell type (lines 243-246 on page 7, Fig. S9; lines 296-313 on pages 8-9, Fig. S11). We also followed your comment on “limited to brain tissue” and extended our analysis to two new 10X Visium datasets obtained from human heart and ovarian cancer (details are provided in the response to your comments #3 and #4 below).*

*We hope the above fully addresses your general concern. We would also be more than happy to accommodate any additional analyses you would like to suggest that are reasonable for a method paper and that would further help with biological interpretation and improve the quality of our manuscript.*

Specific Comments:

1.	The authors should reconsider the name of the tool:

https://www.dictionary.com/browse/expediate.

*Response: Thank you and sorry for the typo. It should be “expedited” instead of “expediated”. The naming convention of our method follows that of the popular bioinformatics method EMMAX[26] (EMMA eXpedited; Nature Genetics 2010), which is a scalable extension of EMMA[27] (Efficient Mixed-Model Association; Genetics 2008).*

2.	The phrase "dozens to thousands of GB physical memory" is not appropriate. Also some people use physical memory as to mean both RAM and hard drives (like here: Difference Between Physical and Virtual Memory | Compare the Difference Between Similar Terms). So maybe "physical RAM memory" on this first instance to clarify this.

*Response: Thank you. Following your suggestion, we have added “RAM” inside “physical memory” to form “physical RAM memory” throughout the text.*

3.	Although the authors provide a proper evaluation of their methods on the main current ST platforms, they limit themselves to using only brain samples. It would be really beneficial for their work to include other tissues that are also publicly available.

4.	The authors need to include an example using 10X Genomics Visium data, which is becoming more widely used due to its commercialization.

*Response: Thank you for both comments. Following your suggestions, we have performed new analyses on two newly added datasets from 10X Visium: one on human ovarian cancer obtained using the targeted Visium spatial technique and the other on human heart obtained using the whole transcriptome Visium spatial technique. The new results are presented in the Figs. S12 and S13, with data processing details provided in the Materials and Methods section (lines 680-686 on page 15). The results on both data are consistent with our main conclusions that SPARK-X produced calibrated P values under permuted null, detected more SE genes than SPARK, SPARK-G and SpatialDE, while being orders of magnitude fasters and using orders of magnitude less memory than the three other methods. We also validated the SE genes detected by SPARK-X in these datasets through functional enrichment analysis and visual inspection of their spatial expression patterns (Fig. S13). Because the new results are consistent with existing conclusions and because all existing 10X Visium data are currently still of moderate size, we decided to describe these new results in the Discussion section (lines 342-350 on page 10).*

5.	The line numbering restarts on each page, which makes the reviewing process a bit difficult.

*Response: Thank you. We have switched to continuous line numbering.*

6.	The authors' statement "trendsceek[4], SpatialDE[5], and SPARK[6]. Unfortunately, even the latter two computationally efficient methods are not readily applicable for analyzing large-scale sparse spatial transcriptomic data", relies on trendsceek being known as computationally inefficient - is this backed up by any evidence?

*Response: Yes, this statement is supported by both the Giotto paper [28] and the SPARK paper [2]. Both publications mentioned that trendsceek is at least ten times slower than the other two methods. Following your comment, we added new analysis by applying trendsceek to analyze the simulated data. We found that trendsceek incurs heavy computational burden even in the simulations with the smallest sample size (n=300). In particular, it took trendsceek 2,423 minutes to analyze 10,000 genes there. In contrast, it only took SPARK, SPARK-G, SpatialDE and SPARK-X, 40 minutes, 5 minutes, 7 minutes and 27 seconds, respectively, to analyze the same data. Therefore, trendsceek is at least 60 times slower than SPARK and 5,000 times slower than SPARK-X and are not applicable to simulations with larger samples. We have added the aforementioned two citations to support that sentence in the introduction (line 47 on page 2). In addition, we have added a few sentences in the Materials and Methods section to mention the observed computational burden of trendsceek in the simulations (lines 730-733 on page 19).*

7.	Can the authors comment on what Bayesian alternatives could be used as an alternative to p-values?

*Response: The Bayesian alternative to hypothesis testing is model comparison and the Bayesian alternative to p-value is the Bayes factor. Some well-known comments on Bayes factor and Bayesian inference in the statistics literature can be found in these classical papers (among many others) [29, 30]. Because no Bayesian approach has been developed for detecting spatially expressed genes in spatial transcriptomics studies, we only listed the two references here for your convenience but did not include the comments from these references to the updated manuscript.*

8.	Spatial expression patterns include hotspot, streak, and gradient. It might be worth writing a comment (in the discussion perhaps?) about how much this covers geometrical patterns found in real images and to what extent other patterns (like circular gradient) would be picked up.

*Response: Thank you for the comment. We primarily followed Trendsceek [31] and SPARK [2] to use these three spatial expression patterns (hotspot, streak and gradient) in the simulations. While these patterns are relatively simple, they are the building blocks that can be used to generate various complex geometrical patterns found in the real datasets. In addition, many genes we identified in the real data did display some of these simple patterns. For example, Dlg2 shows enriched expression in focal areas (Fig.4). Kctd12 shows enriched expression in a streak area (Fig.4). Gphn displays a gradient pattern (Fig.4). While we only used simple patterns in the simulations, we note that SPARK-X did detect a range of complex patterns in the five real datasets more easily than the other methods. Indeed, a key methodological feature of SPARK-X is its ability to incorporate a range of kernels to optimally detect a wide variety of possible spatial expression patterns potentially encountered in real datasets. We have added comments on this important issue in the updated Discussion section (lines 377-388 on page 11) and Method overview section (lines 489-494 on page 14; lines 515-521 on pages 14-15).*

*In addition, we followed your suggestion and simulated data with a circular gradient pattern. The results are shown below for your convenience (Rev1. Figure 4). The new simulation results are consistent with the main conclusions drawn based on the three patterns explored in the previous simulations and suggest that SPARK-X has higher power than the other methods.*

*Rev1. Figure 4. Power comparison of four different SE methods in detecting the circular gradient pattern in simulations. (A) A representative gene displays the circular gradient spatial expression pattern in the simulation. Color represents relative gene-expression levels (green, high; antique-white, low). (B) Power plot shows the proportion of true positives (y axis) detected by different methods across a range of sample sizes (x axis) for the alternative simulations with moderate sparsity at an FDR cutoff of 0.05. Simulations were performed under moderate fraction of marked cells (30% cells displaying expression gradient) and moderate dispersion (0.2). (C) Power comparison of different methods in the alternative simulations under different fractions of marked cells. Simulations were performed under moderate sample size (n=1,000) and moderate dispersion (0.2). (D) Power comparison of different methods in the alternative simulations under different dispersions. Simulations were performed under moderate sample size (n=1,000) and moderate fraction of marked cells (0.3).*

*References:*

*1.	Svensson V, Teichmann SA, Stegle O. SpatialDE: identification of spatially variable genes. Nat Methods. 2018;15(5):343-6.*

*2.	Sun S, Zhu J, Zhou X. Statistical analysis of spatial expression patterns for spatially resolved transcriptomic studies. Nat Methods. 2020;17(2):193-200.*

*3.	Broadaway KA, Cutler DJ, Duncan R, Moore JL, Ware EB, Jhun MA, et al. A statistical approach for testing cross-phenotype effects of rare variants. The American Journal of Human Genetics. 2016;98(3):525-40.*

*4.	Wessel J, Schork NJ. Generalized genomic distance–based regression methodology for multilocus association analysis. The American Journal of Human Genetics. 2006;79(5):792-806.*

*5.	Wu MC, Lee S, Cai T, Li Y, Boehnke M, Lin X. Rare-variant association testing for sequencing data with the sequence kernel association test. The American Journal of Human Genetics. 2011;89(1):82-93.*

*6.	Lin H-T, Lin C-J. A study on sigmoid kernels for SVM and the training of non-PSD kernels by SMO-type methods. submitted to Neural Computation. 2003;3(1-32):16.*

*7.	Fan J, Heckman NE, Wand MP. Local polynomial kernel regression for generalized linear models and quasi-likelihood functions. Journal of the American Statistical Association. 1995;90(429):141-50.*

*8.	Antoniadis A, Paparoditis E, Sapatinas T. A functional wavelet–kernel approach for time series prediction. Journal of the Royal Statistical Society: Series B (Statistical Methodology). 2006;68(5):837-57.*

*9.	Barla A, Odone F, Verri A, editors. Histogram intersection kernel for image classification. Proceedings 2003 international conference on image processing (Cat No 03CH37429); 2003: IEEE.*

*10.	Wu J, Rehg JM, editors. Beyond the euclidean distance: Creating effective visual codebooks using the histogram intersection kernel. 2009 IEEE 12th International Conference on Computer Vision; 2009: IEEE.*

*11.	Boughorbel S, Tarel J-P, Boujemaa N, editors. Generalized histogram intersection kernel for image recognition. IEEE International Conference on Image Processing 2005; 2005: IEEE.*

*12.	Genton MG. Classes of kernels for machine learning: a statistics perspective. Journal of machine learning research. 2001;2(Dec):299-312.*

*13.	Vishwanathan S, Smola AJ. Fast kernels for string and tree matching. Kernel methods in computational biology. 2004;15:113-30.*

*14.	Sahami M, Heilman TD, editors. A web-based kernel function for measuring the similarity of short text snippets. Proceedings of the 15th international conference on World Wide Web; 2006.*

*15.	Srebro N, editor How good is a kernel when used as a similarity measure? International Conference on Computational Learning Theory; 2007: Springer.*

*16.	Nguyen HV, Bai L, editors. Cosine similarity metric learning for face verification. Asian conference on computer vision; 2010: Springer.*

*17.	Norouzi M, Fleet DJ, Salakhutdinov RR, editors. Hamming distance metric learning. Advances in neural information processing systems; 2012.*

*18.	Danielsson P-E. Euclidean distance mapping. Computer Graphics and image processing. 1980;14(3):227-48.*

*19.	Anders S, Huber W. Differential expression analysis for sequence count data. Nature Precedings. 2010:1-.*

*20.	Love MI, Huber W, Anders S. Moderated estimation of fold change and dispersion for RNA-seq data with DESeq2. Genome biology. 2014;15(12):1-21.*

*21.	Tiberi S, Robinson MD. BANDITS: Bayesian differential splicing accounting for sample-to-sample variability and mapping uncertainty. Genome biology. 2020;21(1):1-13.*

*22.	Harrison PF, Pattison AD, Powell DR, Beilharz TH. Topconfects: a package for confident effect sizes in differential expression analysis provides a more biologically useful ranked gene list. Genome biology. 2019;20(1):1-12.*

*23.	Chen W, Li Y, Easton J, Finkelstein D, Wu G, Chen X. UMI-count modeling and differential expression analysis for single-cell RNA sequencing. Genome biology. 2018;19(1):1-17.*

*24.	Korthauer KD, Chu L-F, Newton MA, Li Y, Thomson J, Stewart R, et al. A statistical approach for identifying differential distributions in single-cell RNA-seq experiments. Genome biology. 2016;17(1):1-15.*

*25.	Vallejos CA, Richardson S, Marioni JC. Beyond comparisons of means: understanding changes in gene expression at the single-cell level. Genome biology. 2016;17(1):1-14.*

*26.	Kang HM, Sul JH, Service SK, Zaitlen NA, Kong S-y, Freimer NB, et al. Variance component model to account for sample structure in genome-wide association studies. Nature genetics. 2010;42(4):348-54.*

*27.	Kang HM, Zaitlen NA, Wade CM, Kirby A, Heckerman D, Daly MJ, et al. Efficient control of population structure in model organism association mapping. Genetics. 2008;178(3):1709-23.*

*28.	Dries R, Zhu Q, Dong R, Eng C-HL, Li H, Liu K, et al. Giotto: a toolbox for integrative analysis and visualization of spatial expression data. Genome biology. 2021;22(1):1-31.*

*29.	Kass RE, Raftery AE. Bayes factors. Journal of the american statistical association. 1995;90(430):773-95.*

*30.	Efron B. Why isn't everyone a Bayesian? The American Statistician. 1986;40(1):1-5.*

*31.	Edsgard D, Johnsson P, Sandberg R. Identification of spatial expression trends in single-cell gene expression data. Nat Methods. 2018;15(5):339-42.*

**Second round of review**

**Reviewer 2**

Re: Introductory remarks about lack of new biological knowledge.

I think their response is convincing, particularly given that it is a Methods paper (and the conditions associated with that), and the editor’s comment in their favour.

Comment 1 - Renaming:

They have made the change I suggested so I am obviously satisfied with this one.

Comment 2 - physical memory:

They have made a reasonable change.

Comments 3 & 4 - Adding non-brain data, and adding Visium data:

It seems that they have performed extra analysis in line with what was desired.

Comment 5 - Line numbering:

They have switched to continuous line numbering, so this is fine.

Comment 6 - Information about trendsceek:

They have referenced and added convincing evidence for their implied claim about trendsceek being slow - this is satisfactory.

Comment 7 - Bayesian alternative:

The authors are basically saying that this comment isn’t relevant as no-one has done a Bayesian approach. I am fine with this.

Comment 8 - Different spatial patterns:

Their counterargument is:

It does detect more complicated spatial patterns than the toy examples, and they have updated the Methods and Discussion to talk about this. Circular gradient analysis is good too. Most importantly though, the comments in reply to Reviewer 1, Comment 1 about the 10 transformations are relevant here. Their reply to that and associated modifications to the manuscript explain more clearly that they can detect a wide range of patterns. Overall, this is satisfactory and has improved the manuscript.

Recommendation:

I think the authors have done a good job addressing our main overall concern (novel biology), and have made minor improvements to the manuscript in line with all reviewers’ comments. Overall I’m happy that they expanded their analyses to other tissues and they now included Visium as a supported technology. I am satisfied to recommend it for publication.
